# Assessing the potential for insecticide cross‐resistance to a novel insecticide in the Colorado potato beetle ( *Leptinotarsa decemlineata* ) using phenotypic and transcript expression analysis

**DOI:** 10.1002/ps.71027

**Published:** 2026-06-18

**Authors:** Emma E. Terris, Benjamin Z. Bradford, Faith Kulzer, Justin Clements, Kurt H. Lamour, Sean D. Schoville, Russell L. Groves

**Affiliations:** ^1^ Department of Entomology University of Wisconsin‐Madison Madison WI USA; ^2^ Department of Entomology and Plant Pathology University of Tennessee Knoxville TN USA

**Keywords:** Colorado potato beetle, cross‐resistance, imidacloprid, insecticide resistance, isocycloseram, metabolic detoxification

## Abstract

**BACKGROUND:**

Cross‐resistance in the Colorado potato beetle (*Leptinotarsa decemlineata*) threatens insecticide efficacy and sustainable pest management. While metabolic detoxification is often implicated in resistance, its role in driving cross‐resistance evolution to novel insecticides remains unclear. Here, we evaluated phenotypic and transcriptional responses to both the widely used neonicotinoid imidacloprid and the novel and recently registered insecticide isocycloseram across multiple *L. decemlineata* populations within the United States. We tested for pre‐existing genetic variation and expression patterns linked to detoxification pathways.

**RESULTS:**

Several populations showed significant phenotypic resistance to imidacloprid, while all populations remained more susceptible to isocycloseram. However, positive correlations between the responses to imidacloprid and isocycloseram provide evidence for emerging phenotypic cross‐resistance. Transcript expression analysis revealed significant, but inconsistently patterned treatment group‐, population‐, and dose‐based responses. Inducible up‐regulation of transcript expression was observed only in the imidacloprid treated samples.

**CONCLUSION:**

These findings support the presence of phenotypic cross‐resistance in *L. decemlineata*, however, transcriptomic analyses of a subset of metabolic detoxification genes did not reveal consistent expression patterns across populations, treatments, or doses, limiting definitive inference about the contribution of detoxification pathways to this response. Variation seen here in gene expression across populations underscores the complexity of resistance evolution and the need for further investigation into the mechanisms underlying cross‐resistance. © 2026 The Author(s). *Pest Management Science* published by John Wiley & Sons Ltd on behalf of Society of Chemical Industry.

## Graphical abstract

Phenotypic and transcriptional responses to imidacloprid and isocycloseram were examined across U.S. populations of the Colorado potato beetle, showing evidence of phenotypic cross‐resistance alongside population‐level variation in detoxification gene expression.

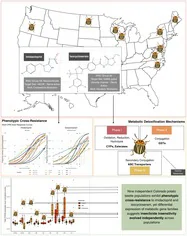

## 1 INTRODUCTION

The evolution of cross‐resistance to insecticides in pest species remains a critical area of research despite extensive investigation into the molecular pathways.^1^ Cross‐resistance poses a substantial challenge for effective and durable pest control through pesticide use, due to the overlapping resistance mechanisms that have been documented in several insect pest species.^2^, ^3^, ^4^ This phenomenon, defined as resistance to insecticides in the absence of prior exposure, complicates management strategies and suggests the involvement of shared molecular mechanisms driving cross‐resistance.^5^ Although cross‐resistance is typically associated with insecticides using the same mode of action (MoA), it can also develop through various other molecular mechanisms, including metabolic detoxification, target‐site mutations, and diminished cuticular penetration.^6^, ^7^

In particular, metabolic detoxification has been proposed as a possible major contributor to cross‐resistance among insecticides.^8^, ^9^, ^10^ This process begins with Phase I metabolism, involving oxidation, hydrolysis, and reduction reactions that introduce functional groups for xenobiotic breakdown. Key enzymes in this phase, such as cytochrome P450 monooxygenases (CYPs) and esterases, are essential for detoxification in many insects.^2^, ^4^, ^11^, ^12^ Phase II and Phase III metabolism subsequently facilitate conjugation and transport of xenobiotic metabolites, respectively, enhancing their solubility and promoting transmembrane export. Enzymes central to these processes, including glutathione S‐transferases (GSTs) and ATP‐binding cassette (ABC) transporters, play critical roles in the excretion of metabolic products and have been widely implicated in insecticide resistance.^5^, ^12^, ^13^, ^14^ The ability of these detoxification enzymes, and the functional redundancy in the gene families encoding them, to neutralize a diverse range of xenobiotics can lead to enhanced resistance across multiple insecticides.^5^, ^15^ This broad‐spectrum detoxification mechanism underscores the complexity of managing resistant pest populations and highlights the potentially critical role of metabolic detoxification in the evolution of cross‐resistance.^5^

An intractable agricultural pest, the Colorado potato beetle (*Leptinotarsa decemlineata*, Say) is well known for its ability to rapidly evolve resistance to chemical MoA classes of insecticides.^13^, ^16^ After it was first recognized as a Solanaceous crop pest in 1859, the beetle was effectively managed with insecticides until the first recorded case of resistance to DDT in the mid‐twentieth century.^16^, ^17^ Since then, significant insensitivity has been documented against most major compounds, spanning at least 37 different insecticidal classes, as well as multiple target sites and MoAs.^18^, ^19^, ^20^ Previous evaluations of genetic variation patterns in the beetle have revealed geographical population structure and an abundance of genetic diversity among populations across the United States.^21^, ^22^ Even within local populations, there is a diverse range of both insecticide resistance levels and expression across insecticide resistance linked genes.^13^, ^23^, ^24^

In the current study, patterns of gene expression in *L. decemlineata* were investigated following exposure to imidacloprid, a frequently used insecticide, and isocycloseram, a novel compound with a distinct target site and mode of action (MoA Class 30) and target site. Due to its novelty, we hypothesized that most beetle populations would be highly susceptible to isocycloseram, and significantly less susceptible to imidacloprid, particularly in populations from the eastern and midwestern U.S. where resistance has been widely reported.^2^, ^25^ Building upon this hypothesis, as well as evidence that contemporary insecticide resistance typically involves polygenic genetic architecture^25^, ^26^, ^27^, ^28^ and rapid evolutionary responses driven by independent selection across geographically distinct populations,^13^, ^14^ we examined differential gene transcript expression to test three additional hypotheses: (i) expression patterns of metabolically linked genes would be similar in response to both insecticides, (ii) transcript expression would vary among populations according to their existing resistance status, and (iii) populations exhibiting elevated phenotypic insensitivity would demonstrate constitutive transcript expression across doses, whereas susceptible populations would display inducible gene expression.^22^, ^29^

## 2 MATERIALS AND METHODS

### 2.1 *Leptinotarsa decemlineata* collection and insecticides

Overwintered adult *L. decemlineata* were requested from several field locations, listed in Tables S1 and S2, around the continental U.S. in the late spring and early summer of 2020 and 2021 for leaf‐dip bioassays against two test substances/active ingredients (imidacloprid (Admire® Pro Systemic Protectant) and isocycloseram (marketed under Syngenta's Plinazolin® technology platform)).

Imidacloprid is a commonly used neonicotinoid and a member of the Group 4A, mode of action insecticide class. As a fast acting, nerve targeting group, neonicotinoids are competitive modulators of the nicotinic acetylcholine receptor (nAChR) in the beetle nervous system (IRAC classification Group 4A).^19^ Registered for use in 1995, imidacloprid became a key insecticide for potato cultivation, effectively targeting pests such as the *L. decemlineata* and various sucking insect pests including aphids and leafhoppers (Bayer Crop Sciences, North America, St. Louis, MO).^30^

Isocycloseram is a newly registered insecticide that inhibits the Gamma‐aminobutyric acid (GABA) gated chloride channel function in the nervous system (IRAC MoA classification Group 30), which very recently received (registered 20 November 2025 for first grower purchase and use in the 2026 season as Zivalgo™) full Section 3.3 registration for use against *L. decemlineata* in North America (Syngenta, North America, Greensboro, NC).^19^, ^31^

Approximately 300 adult beetles were collected from field locations by cooperators and mailed overnight in refrigerated containers to the University of Wisconsin, Vegetable Entomology Laboratory. Upon arrival, all adult insects were released into cages containing untreated, 3‐5‐week‐old greenhouse‐grown potato plants for a 72‐96‐h acclimation period in advance of the assay. Adults (*n* = 216–270) from each field‐collected population were subjected to leaf‐dip bioassays to determine estimates of the median lethal dose (LC_50_, the dose necessary to kill 50% of test insects) associated with serial exposures to both imidacloprid and isocycloseram for that population.

### 2.2 Lethal concentration assessments within populations

Following the acclimation period, actively feeding adults were randomly selected from among the test population and divided into nine groups representing the serial dilutions of the test substance, as well as a control of 0 ppm (*n* = 24 individuals per group), with six biological replicates. Serial dilutions in water for imidacloprid measured in parts per million (ppm) were: 0.001, 0.01, 0.1, 1, 10, 100, and 1000. Serial dilutions for isocycloseram measured in ppm included: 0.001, 0.005, 0.01, 0.05, 0.1, 0.5, 1, and 10. Dilution ranges were chosen based on preliminary dose response assays performed in the laboratory.

To begin the bioassay, a terminal trifoliate potato leaf was removed from a fully expanded, greenhouse‐grown potato plant (*Solanum tuberosum* cv. Silverton Russet) for each dilution of the test substance. To ensure consistency over the assay period, the terminal trifoliate leaf was selected to be sufficiently large so as not to limit feeding by the adults prior to the conclusion of the 4‐day bioassay period. Briefly, leaflets were individually dipped in the nine representative dilutions of the respective test substance by grasping the exposed end of a petiole and completely submerging the remainder of the leaflets. Leaflets were allowed to completely dry within a chemical fume hood. In advance of the initial exposure of adults, filter paper was placed in the bottom of 150 × 15 mm Petri dishes. Air‐dried leaflets were then placed into labeled dishes and four *L. decemlineata* adults were transferred using forceps. Adults were allowed to feed on treated foliage for 96 h in an incubator held at 26 °C, 70% humidity, and a light and dark (L:D) cycle of 16:8 h, and a second series of treated foliage containing the same serial dose was provided after 48 h. After 48 and 96 h, the number of live, incapacitated (defined as beetles unable to right themselves when flipped over), and dead adults were recorded. Incapacitated and dead beetles were pooled, and LC_50_ values for each population were calculated and fitted with 95% confidence limits. Abbott's correction was used to normalize the data, and a log_10_ Probit regression analysis was performed in R (v4.3.2) using the ecotox package.^32^, ^33^ Surviving beetles from every dose in the 48‐h assays were flash frozen in liquid nitrogen and stored at −80 °C for downstream genetic analyses.

Statistical analyses were conducted in R to assess the potential for phenotypic cross resistance. Shapiro–Wilk tests were used to evaluate the normality of log‐transformed LC values for imidacloprid and isocycloseram. Due to significant data deviation from normality (*P* < 0.001), we used non‐parametric methods for correlation and variance analysis. Spearman's rank correlation and Kendall's tau were calculated to assess the monotonic association between the dose–response values of both insecticides across all populations, as well as calculate LC levels (LC_20_, LC_50_, LC_90_). To test for homogeneity of variance, we performed Levene's test using median‐centered groupings. An aligned rank transform ANOVA (ART ANOVA; art function, ARTool package)^34^ was used to test the main and interaction effects of insecticide and population on dose response, allowing for factorial analysis under non‐parametric assumptions. *Post hoc* pairwise comparisons were conducted using estimated marginal means (emmeans package),^35^ with contrasts performed between insecticides within each population. Significance thresholds were set at *α* = 0.05, and *P*‐values were adjusted using Benjamini‐Hochberg procedure for multiple comparisons where applicable. In order to provide a basis for direct phenotypic comparison, resistance ratios (RR_50_) at the median lethal concentrations (LC_50_) were calculated by dividing LC_50_ values of test populations by the LC_50_ of a susceptible lab‐reared colony for each insecticide.

### 2.3 Sequencing, quantification, and quality control

Total RNA was extracted for sequencing following select pesticide exposures for each population, which were chosen using estimated LC_20_ and LC_50_ values for each substance. Approximate doses that most closely matched LC_20_ and LC_50_ values for respective populations were chosen to reflect the induced genetic response to both insecticides, while avoiding extreme cellular toxicosis. For imidacloprid, extracted dilutions included 0, 0.1, and 10 ppm, with the addition of 100 ppm for two more phenotypically resistant populations (NY and ME). For isocycloseram, extracted dilutions included 0 ppm, 0.005 ppm, 0.5 ppm for all beetle populations. Total RNA was extracted from adult beetles (*n* = 6) at each chosen dilution using Trizol (Invitrogen) and the quality and quantity of RNA was measured using a Nanodrop spectrophotometer (Thermo Fisher Scientific, Wilmington, DE). Extracted RNA samples were equalized using Qubit Fluorometric Quantification system and converted to cDNA using SuperScript III kits (Invitrogen). A total of 20 μL of beetle cDNA for each biological replication (*n* = 6) was submitted to Floodlight Genomics LLC (https://floodlightgenomics.com/) for targeted amplicon sequencing. Gene targets for the amplification (*n* = 299) were chosen based on data from previous studies that identified candidate resistance genes associated with metabolic detoxification and target site resistance. Gene families on this list include, but are not limited to, cytochrome p450s, GSTs, ABC transporters, and esterases.^5^, ^13^, ^14^ Custom primer sequences were designed to specific transcripts of interest using the Primer‐BLAST tool.^36^ The candidate gene IDs, descriptions, and primer sequences are provided in Table S3. Select housekeeping genes (*n* = 11 of 299) were chosen and included as a baseline reference of expression and are listed in Table S4.

Primers and cDNA were provided to Floodlight Genomics for processing using their MonsterPlex workflow. MonsterPlex, an optimized amplicon sequencing approach, includes triplicate PCR amplification of each biological replicate to minimize variation and sequencing on the Illumina platform following the manufacturer's directions. Floodlight Genomics delivered sample‐specific raw transcript sequence reads, which were aligned to annotated sequences. The data were sequenced using the NovaSEQ X Plus device with a 2 × 150 bp configuration, merging all paired reads during processing to enhance coverage, which ranged from approximately 100 000 reads to over 1 000 000 per sample. A replicated library of samples was also provided by Floodlight Genomics for quality control. Targeted transcript expression data generated for this study were deposited in the NCBI Sequence Read Archive (SRA) under BioProject accession PRJNA1400069.

The quality of the raw cDNA amplicon sequencing reads was assessed using MultiQCv1.23^37^ and then trimmed using Cutadapt v1.18^38^ to improve the quality of analysis. Salmon v1.10.2^39^ was then employed for sequencing data analysis to quantify transcript abundances, accounting for sample‐specific parameters and biases. Using a quasi‐mapping approach, Salmon processes raw sequencing reads directly without generating intermediate alignment files, enhancing efficiency and reducing computational requirements.^39^ However, to further increase read coverage, pre‐computed alignments were first generated using HISAT2 v2.2.1^40^ by mapping to the *L. decemlineata* reference genome (NCBI Refseq accession: GCF_000500325) and then processed by Salmon to estimate read counts.

### 2.4 Analysis

In order to test whether metabolically linked genes responded similarly to imidacloprid and isocycloseram, transcript abundance estimates were generated with Salmon and imported into R using the tximport package,^41^ which aggregates transcript‐level counts to the gene level while correcting for transcript length and inferential variance. Technical replicate consistency was confirmed by calculating the differences in gene counts between replicates; the grand mean (0.0005932173) and standard deviation (0.0005131804) were deemed sufficiently low to collapse replicates.

Differential gene expression analyses were performed using DESeq2 (v1.38.3).^42^ Transcript‐level abundance estimates were imported with tximport and aggregated to the gene level to construct a DESeqDataSet with chemical treatment, geographic location, and dose as main effects (~ chemical + location + dose_ppm).^42^ Biological replicates were collapsed prior to model fitting, and counts were normalized and modeled using a negative binomial framework.

Wald tests from the full model were used to estimate effect sizes and directionality of differential expression, including pairwise contrasts between chemical treatments and controls and the continuous dose effect. Statistical significance was assessed using Benjamini‐Hochberg false discovery rate (FDR) correction with an adjusted *P*‐value threshold of 0.05. Likelihood ratio tests (LRTs) were conducted by comparing the full model to reduced models excluding chemical, dose, or location to assess the overall contribution of each factor to gene expression variation. Genes with significant LRT results were considered responsive to the tested factor after accounting for covariates. Variance‐stabilized expression values (VST) were used for clustering and visualization of gene expression patterns across samples.

To test whether gene expression patterns varied among populations according to their existing resistance status, we conducted principal component analyses (PCA) of VST counts using plotPCA() in DESeq2, visualized with ggplot2 and ggforce, with sample clusters framed using geom_mark_hull. To statistically assess population, treatment, dose, and their interactions, we conducted PERMANOVA^43^ using the adonis2 function in vegan^44^ with 999 permutations applied to Euclidean distance matrices calculated from the first two principal components (PC1 and PC2) of VST data. Populations with insufficient replication for within‐population comparisons (North Carolina, NC) were excluded.

To test whether resistant populations exhibit constitutive expression while susceptible populations show inducible responses, VST normalized DESeq2 counts were subset by regulation direction (up‐ *vs*. down‐regulated relative to controls) and chemical treatment (imidacloprid *vs*. isocycloseram). For each population × chemical × insecticide dose, normalized counts were merged with sample metadata using dplyr (v1.1.3) and tidyr (v1.3.0). Expression values were log10‐transformed [log10(expression +1)] to stabilize variance. Within each population, pairwise comparisons between dose levels were performed using Wilcoxon rank‐sum tests via stat_compare_means() in ggpubr (v0.6.0). Statistical significance was annotated based on unadjusted *P*‐values (*P* < 0.05 = *, < 0.01 = **, < 0.001 = ***). Populations with fewer than two replicates per dose level (NC) were excluded from this analysis.

Gene family assignments were determined using EggNOG‐mapper v2.1.12.^45^ The 299 amplicon sequences were annotated against the EggNOG 6.0 database to identify orthologous groups (OGs). Gene family classification was based on COG/KOG orthologous group assignments and manual sorting to eliminate name discrepancies. The groupings represent evolutionarily conserved gene families across different taxonomic levels. Each gene was assigned to its corresponding orthologous group based on sequence similarity and phylogenetic relationships within the EggNOG framework.

## 3 RESULTS

### 3.1 Phenotypic assay

*Leptinotarsa decemlineata* responses to imidacloprid and isocycloseram was measured across 11 populations from across the U.S. LC values revealed varying levels of insensitivity to both insecticides across all populations, with greater resistance shown in response to imidacloprid (Table 1, Fig. S1). In most cases, LC values for imidacloprid were significantly higher than those for isocycloseram, with non‐overlapping 95% confidence intervals indicating statistically greater resistance to imidacloprid (*P* < 0.05). Populations from New York (NY) and Maine (ME) exhibited LC_50_ values for imidacloprid exceeding 100‐fold those of the most susceptible populations, suggesting elevated insensitivity in these populations. In contrast, LC_50_ values for isocycloseram remained lower and statistically similar across populations, indicating limited pre‐existing insensitivity and greater baseline susceptibility. Slope and chi‐square values further support differences in population response homogeneity.

**Table 1:** Probit regression estimates of lethal concentrations (LC_50_) for *L. decemlineata* populations exposed to Imidacloprid and Isocycloseram

		LC_x_ (ppm) ± (95% CI) for Imidacloprid		LC_x_ (ppm) ± (95% CI) for Isocycloseram	
Population	LC_x_	48 h	96 h	48 h	96 h
AARS	50	2.83 (0.914–8.756)	2.24 (0.683–7.376)	1.53 (0.917–2.55)	*0.933 (0.416–2.09)
	RR_50_	10.066	‐	25.929	‐
	*χ* ^2^	*32.0578*	*25.5479*	*37.1846*	*66.0098*
	*Slope*	*0.5709*	*0.5463*	*1.3464*	*0.7959*
AARS (2nd gen)	50	6.31 (3.297–12.057)	3.26 (1.778–5.977)	*0.223 (0.086–0.58)	0.267 (0.164–0.435)
	RR_50_	11.237	‐	31.002	‐
	*χ* ^2^	*27.4232*	*28.4210*	*67.4515*	*54.9876*
	*Slope*	*0.88431*	*0.8843*	*0.6660*	*1.1761*
HARS	50	15.7 (2.995–82.160)	1.12 (0.225–5.600)	2.82 (1.44–5.51)	1.61 (0.769–3.36)
	RR_50_	26.726	‐	47.809	‐
	*χ* ^2^	*51.6739*	*47.9075*	*45.5662*	*55.2763*
	*Slope*	*0.3856*	*0.3822*	*1.0980*	*0.9116*
HARS (2nd gen)	50	0.582 (0.173–1.960)	0.549 (0.191–1.579)	*13.9 (0.381–503)	*0.817 (1.17E‐04‐5730)
	RR_50_	1.092	‐	25.998	‐
	*χ* ^2^	*46.5776*	*49.3693*	*104.1965*	*68.9091*
	*Slope*	*0.4581*	*0.5051*	*0.3266*	*0.1958*
MD	50	3.46 (1.633–7.311)	7.49 (2.987–18.772)	0.154 (0.099–0.239)	0.0783 (0.053–0.116)
	RR_50_	12.004	‐	2.631	‐
	*χ* ^2^	*53.4241*	*57.9992*	*39.2608*	*31.2754*
	*Slope*	*0.6843*	*0.5818*	*1.271*	*1.4409*
ME	50	428 (99.572–1838.459)	436 (59.079–3223.461)	1.44 (0.686–3.03)	1.62 (0.365–7.21)
	RR_50_	58.575	‐	24.483	‐
	*χ* ^2^	*35.6589*	*64.3037*	*60.3178*	*88.3829*
	*Slope*	*0.5135*	*0.3797*	*0.8932*	*0.5270*
NC	50	10.9 (5.064–23.408)	25.2 (9.438–67.366)	0.282 (0.082–0.97)	0.33 (0.157–0.694)
	RR_50_	23.163	‐	2.631	‐
	*χ* ^2^	*40.0426*	*53.7787*	*118.6897*	*84.9647*
	*Slope*	*0.8842*	*0.7612*	*0.5676*	*0.8053*
ND	50	2.18 (0.527–9.036)	0.658 (0.103–4.217)	3.41 (1.6–7.31)	1.2 (0.397–3.61)
	RR_50_	7.550	‐	57.955	‐
	*χ* ^2^	*35.4186*	*36.5387*	*48.0139*	*66.2668*
	*Slope*	*0.4139*	*0.3505*	*0.9893*	*0.6378*
NY	50	629 (164.001–2411.519)	118 (45.661–302.419)	0.0653 (0.003–1.54)	4.13 (0.718–23.7)
	RR_50_	62.361	‐	145.824	‐
	*χ* ^2^	*26.4360*	*49.1588*	*35.8931*	*99.7204*
	*Slope*	*0.6043*	*0.7074*	*0.3339*	*0.4923*
OR	50	36 (18.132–71.382)	*0.559 (0.294–1.064)	0.402 (0.268–0.603)	0.381 (0.236–0.615)
	RR_50_	34.744	‐	6.610	‐
	*χ* ^2^	*26.6181*	*64.2012*	*45.5973*	*55.0427*
	*Slope*	*0.8787*	*0.8026*	*1.5914*	*1.2400*
VA	50	1.95 (1.070–3.558)	1.73 (0.950–3.145)	0.91 (0.55–1.5)	0.783 (0.391–1.57)
	RR_50_	6.459	‐	15.445	‐
	*χ* ^2^	*38.2351*	*32.8809*	*46.2885*	*60.3515*
	*Slope*	*0.8855*	*0.8892*	*1.2837*	*0.8952*

LC_50_ values (ppm) with 95% confidence intervals (CI) given in parentheses for imidacloprid and isocycloseram at two exposure times (48 h and 96 h) across multiple *L. decemlineata* populations. LC_50_ values estimate the concentrations of insecticide required to kill 50% of individuals. Also shown are the resistance ratios at LC_50_ (RR_50_), chi‐square goodness‐of‐fit (*χ* ^2^) statistics and slope values for each probit regression. LC values from models with poor fit (*χ* ^2^ test, *P* < 0.05) were flagged but retained in the analysis. LC_20_ and LC_90_ values (ppm) with 95% confidence intervals (CI) can be found in Table S7.

Abbreviations: AARS, Arlington Agricultural Research Station, Wisconsin; HARS, Hancock Agricultural Research Station, Wisconsin; MD, Maryland; ME, Maine; NC, North Carolina; ND, North Dakota; NY, New York; OR, Oregon; VA, Virginia.

### 3.2 Assessment of phenotypic cross‐resistance

**Figure 1:**
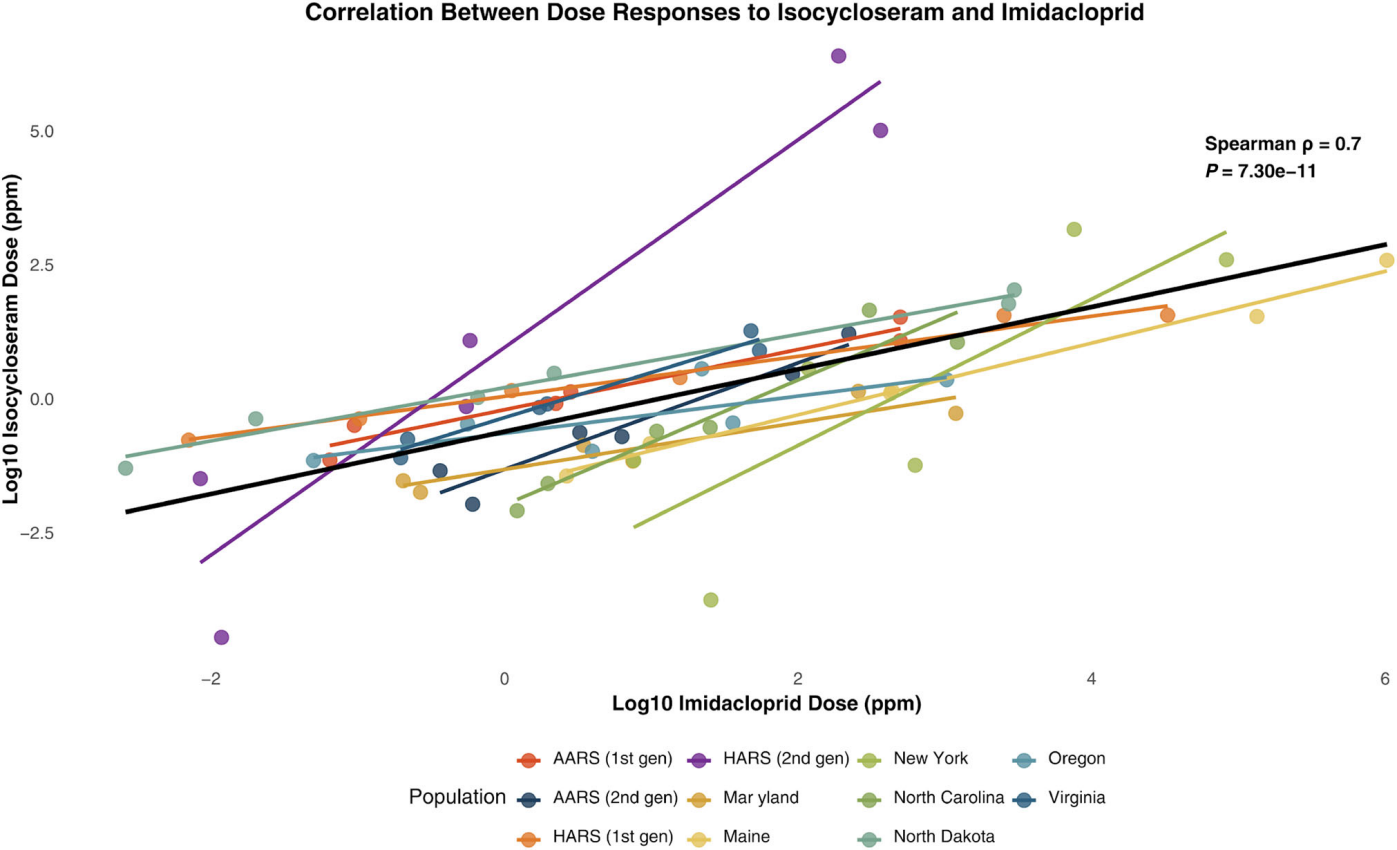
Correlation between dose responses to isocycloseram and imidacloprid. Log‐transformed correlation between dose responses to isocycloseram and imidacloprid across beetle populations. Each point represents a dose–response value for a given population. Colored regression lines indicate population‐specific trends, while the black regression line represents the overall trend across all populations. The Spearman correlation coefficient (*ρ* = 0.7, *P* = 7.30e‐11) indicates a significant positive association between responses to the two insecticides.

A Spearman rank correlation revealed a strong positive association between log‐transformed dose responses to both insecticides across all populations and LC levels (*ρ* = 0.698, *P* < 0.001). Kendall's tau test supported this result (*τ* = 0.512, *P* < 0.001), indicating concordant, dose–response trends across insecticides and potential phenotypic cross‐resistance between the two insecticides (Fig. 1).

To assess variance homogeneity, Levene's test was conducted on the untransformed data, revealing no significant differences in variance across population‐insecticide groups (*P* = 0.378), justifying further factorial analysis. Given the non‐normality of the data, we applied an Aligned Rank Transform (ART) ANOVA to test the effects of insecticide, population, and their interaction. The analysis revealed a significant main effect of insecticide (*F*_1_,_110_ = 131.64, *P* < 0.001) and a significant insecticide × population interaction (*F*_10_,_110_ = 4.27, *P* < 0.001), while the effect of population alone was not statistically significant (*P* = 0.096).

### 3.3 Differential expression analyses

Across all samples, no genes from the 299‐candidate list met the typical threshold for strong differential expression (adjusted *P* < 0.05 and |log_2_ fold‐change| ≥ 2). However, we identified 65 genes that were significantly differentially expressed (adjusted *P* < 0.05), including 33 up‐regulated and 32 down‐regulated genes (Table S5), denoted by positive and negative log‐fold changes, respectively. The candidate gene set targeted the three phases of metabolic detoxification: Phase I enzymes including cytochrome P450 monooxygenases (53 genes) and carboxylesterases (43 genes); Phase II enzymes including glutathione S‐transferases (13 genes); and Phase III transporters including ATP‐binding cassette transporters (42 genes). The remaining 148 genes encompassed diverse cellular functions including ion channels, structural proteins, and other metabolic enzymes. Within treatment groups, control samples exhibited nine up‐ and eight down‐regulated genes; imidacloprid‐treated samples had 16 up‐ and seven down‐regulated genes; and isocycloseram‐treated samples had five up‐ and 15 down‐regulated genes.

Several detoxification‐related genes were among the DEGs. In imidacloprid‐treated samples, these included cytochrome P450 9e2‐like (LOC111507099, p450 family), glutathione S‐transferase 1‐like (LOC111509532, ABC transport family), glutathione S‐transferase D2‐like (LOC111509814, unidentified family), and multiple multidrug resistance‐associated proteins (LOC111502194, ABC transport family; LOC111512003, p450 family; LOC111515719, ASC family). In isocycloseram‐treated samples, DEGs included cytochrome P450s (LOC111504218, p450 family; LOC111508711, p450 family; LOC111508880, Ion transport family; LOC111508881, Carboxylesterase family; LOC111513747, GST family), glutathione S‐transferase 1 (LOC111515122, GST family), venom carboxylesterase‐6‐like (LOC111505753, Carboxylesterase family), esterase E4‐like (LOC111508891, p450 family), liver carboxylesterase 1‐like (LOC111512274, Carboxylesterase family), and multidrug resistance‐associated proteins (LOC111502767, ABC transport family; LOC111515003, GST family; LOC111517473, Neuronal channel ligand‐binding domain family). No identical gene IDs overlapped between the imidacloprid and isocycloseram DEG sets. However, both treatments induced members of the same detoxification gene families.

### 3.4 Analysis of independent factors on genetic response

#### 3.4.1 Insecticide treatment groups

**Figure 2:**
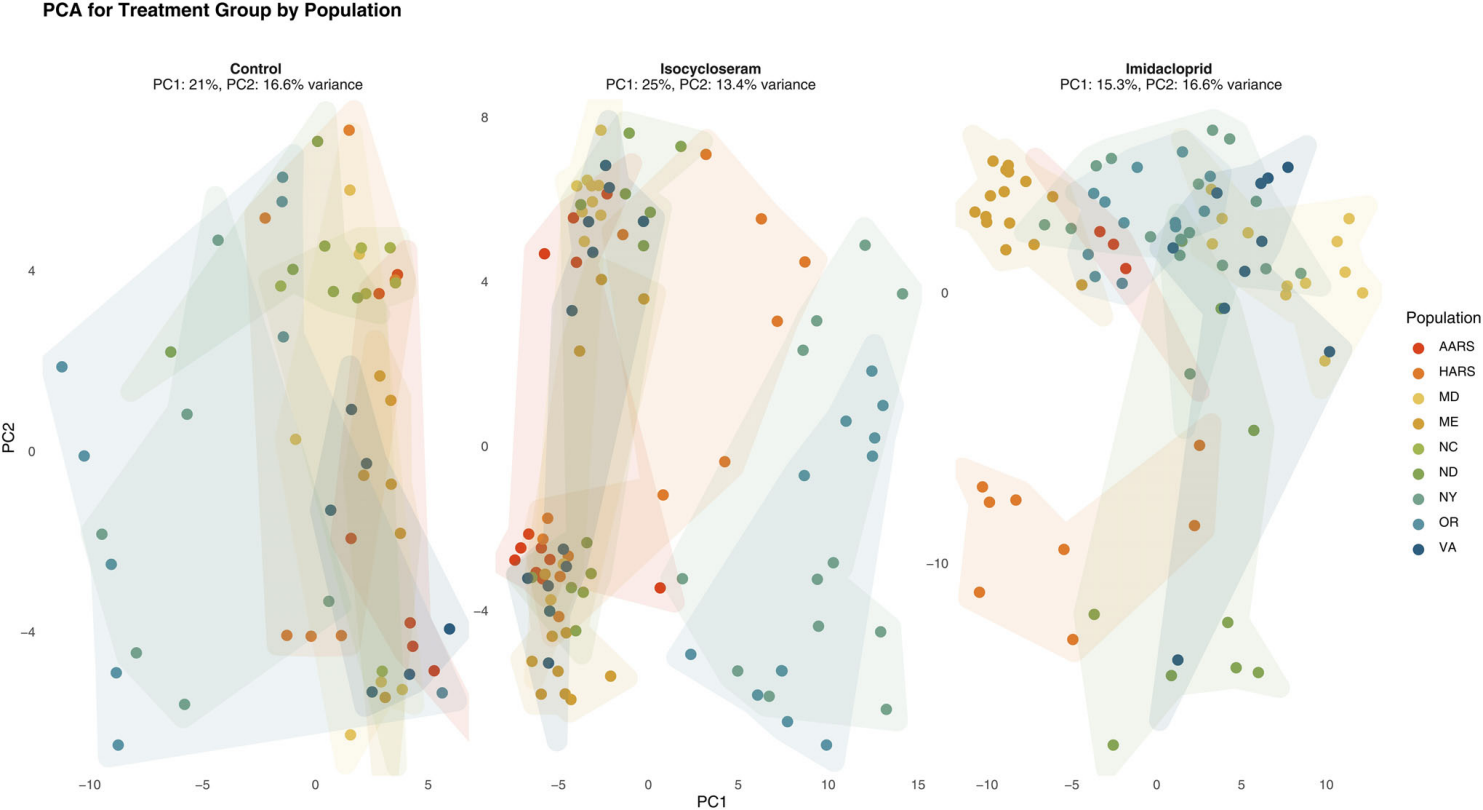
PCA for treatment group by population. Principal component analysis (PCA) of *L. decemlineata* gene expression profiles across all treatments (Control, Isocycloseram, Imidacloprid), with samples colored by population. Shaded polygon boundaries represent population‐level sample dispersion. PC1 and PC2 reported for each treatment group indicate the percentage of variance explained by respective principal components.

Initial comparison of control (untreated) samples revealed substantial baseline transcriptional differences among populations (*R* ^2^ = 0.43, *P* = 0.001), indicating high standing genetic diversity prior to insecticide exposure. A PCA of all samples grouped by insecticide treatment showed partial overlap among populations. Clustering by treatment was limited overall and instead varied in a population‐specific manner, with some populations showing clearer treatment‐driven separation than others (Fig. 2). Despite significant statistical effects of insecticide treatment (*P* = 0.039), population (*P* < 0.001), and their interaction (*P* < 0.001), population clusters were not fully distinct or patterned according to hypotheses.

#### 3.4.2 Population

**Figure 3:**
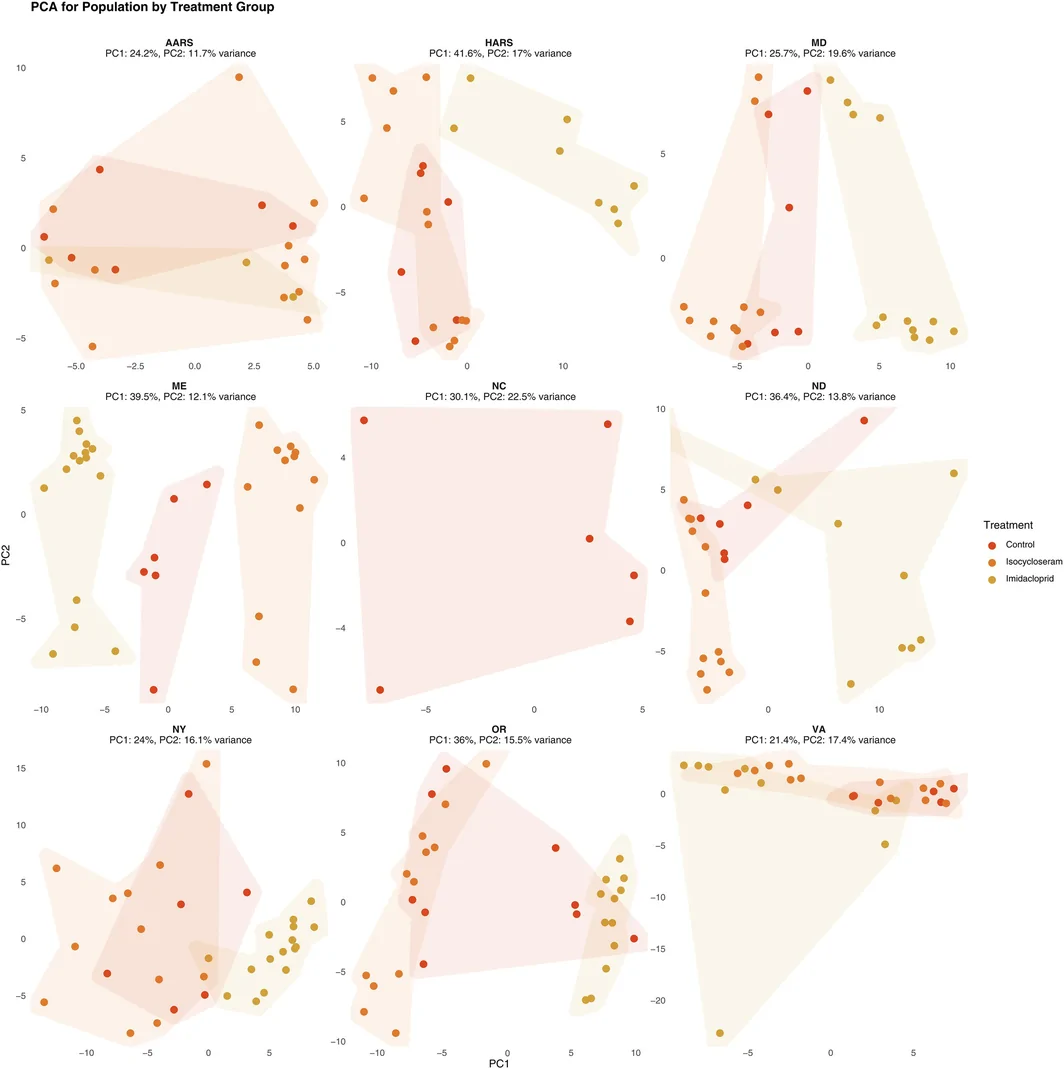
Principal component analysis (PCA) for population by treatment group. PCA of gene expression profiles, sorted by population and colored by chemical treatment (Control, Isocycloseram, Imidacloprid). Shaded polygon regions reflect clusters of samples exposed to each treatment group within a given population. PC1 and PC2 values reflect the percentage of variance captured by the principal components per population. This layout reveals population‐specific expression responses driven by insecticide exposure.

PCA of gene expression in response to increasing imidacloprid doses revealed limited, population‐specific shifts in gene expression (Fig. 3). While treatment groups largely overlapped in PCA space, significant differences in gene expression response among treatments were detected in seven of eight populations (excluding NC from statistical analysis), except for collections from the AARS. The proportion of variance explained by treatment varied widely across populations, ranging from moderate (VA; *R* ^2^ = 0.20, NY; *R* ^2^ = 0.37) to strong (ME, OR, ND, MD, HARS; *R* ^2^ = 0.54–0.68) (Table S6).

#### 3.4.3 Dose of insecticide

**Figure 4:**
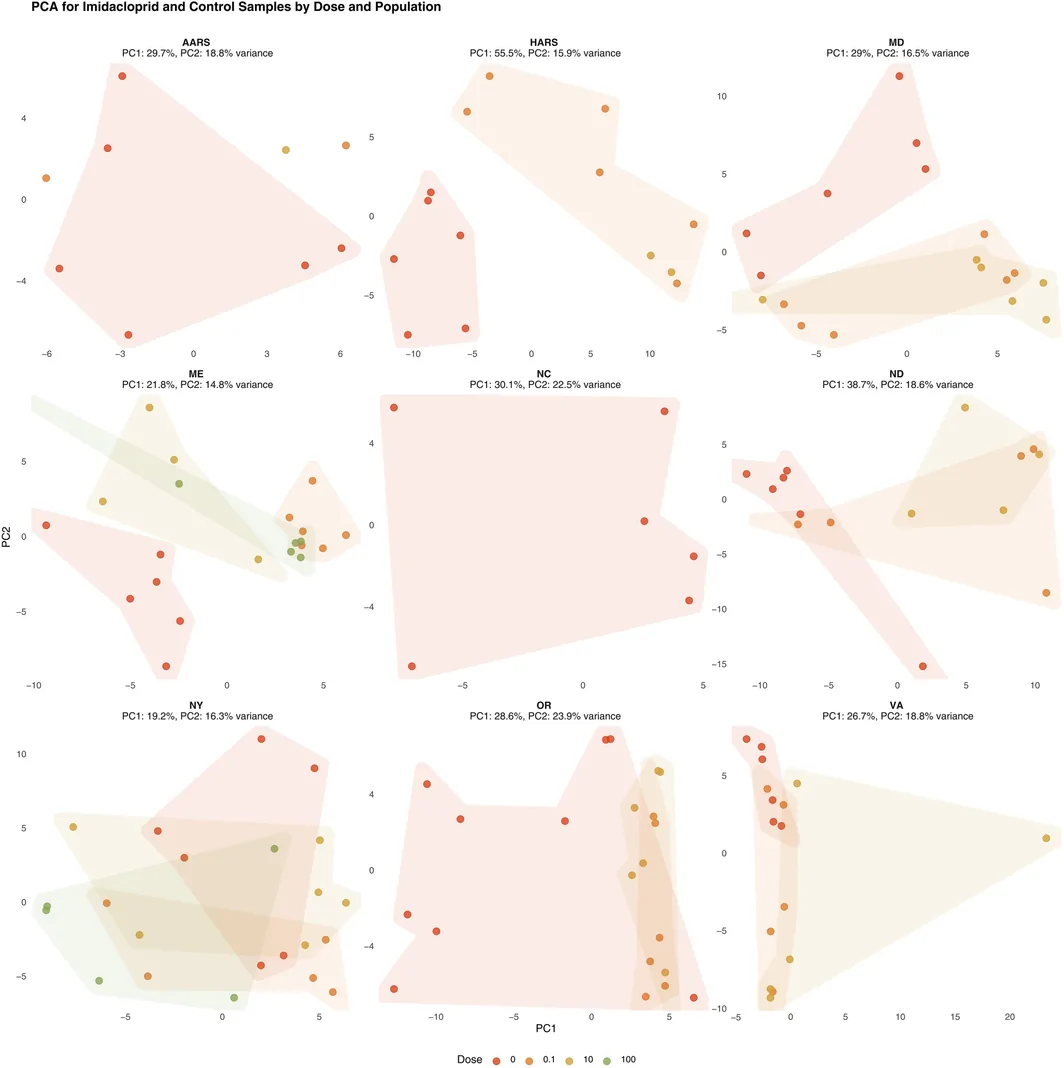
Principal component analysis (PCA) for imidacloprid and control samples by dose and population. PCA plot shows gene expression patterns for *L. decemlineata* exposed to Imidacloprid and Control treatments, grouped by population and colored by dose (0, 0.1, 10, and 100 ppm). Shaded polygon regions represent the clustering of samples from each dose level. This layout reveals population‐specific expression responses driven by different doses of imidacloprid. PC1 and PC2 values reflect the percentage of variance captured by the principal components per population.

PCA of gene expression in response to increasing imidacloprid dose revealed limited and population‐specific gene expression shifts (Fig. 4). The only statistically significant difference among doses occurred in ME (*P* = 0.008, *R* ^2^ = 0.30). Several other populations (HARS, ND, OR, and VA) exhibited marginal trends (*P* = 0.052–0.068), while most showed no detectable effect of dose.

**Figure 5:**
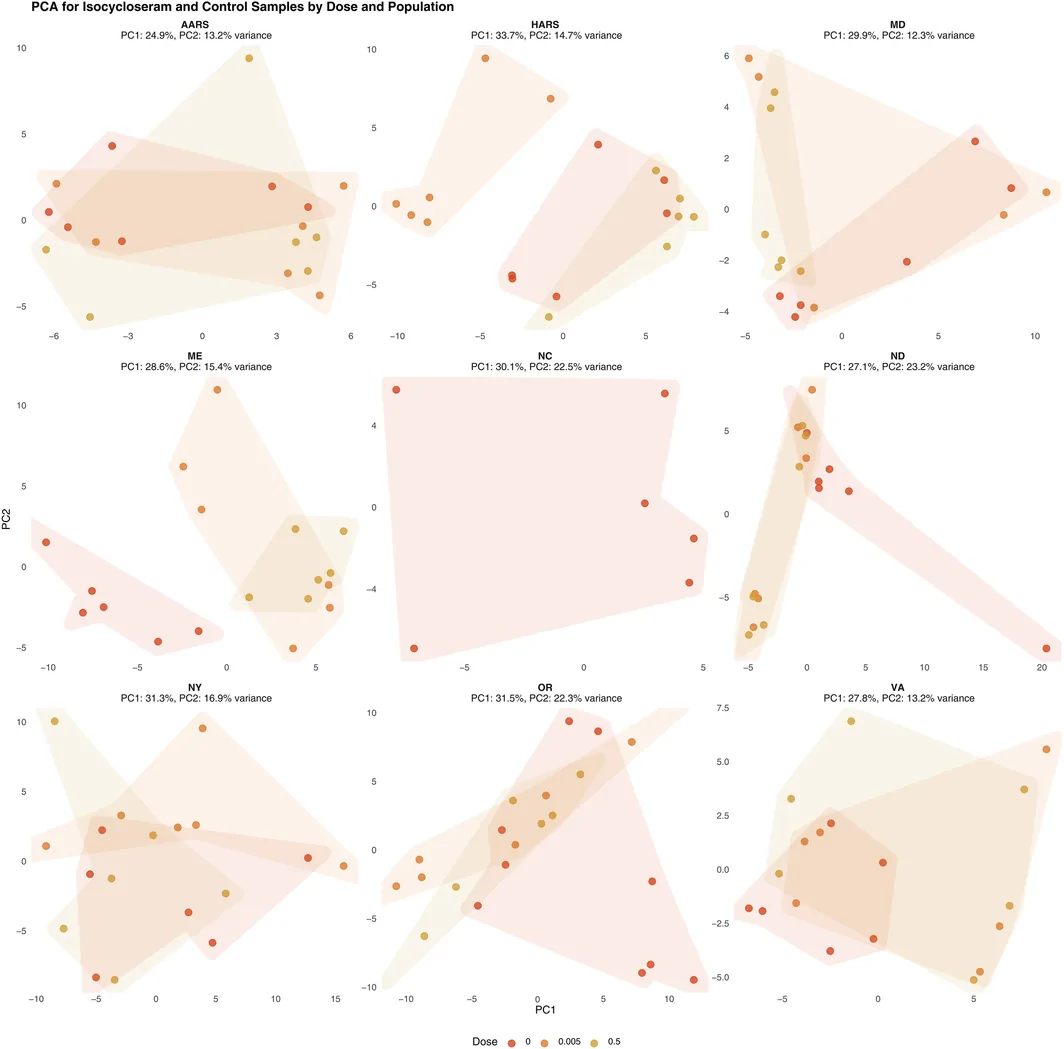
Principal component analysis (PCA) for isocycloseram and control samples by dose and location. PCA plot showing gene expression patterns for *L. decemlineata* exposed to Isocycloseram and Control treatments, grouped by population and colored by dose (0, 0.005, and 0.5 ppm). Shaded polygon regions represent the clustering of samples from each dose level. This layout reveals population‐specific expression responses driven by different doses of isocycloseram. PC1 and PC2 values reflect the percentage of variance captured by the principal components per population.

PCA of gene expression in response to isocycloseram exposure showed little consistent dose‐dependent gene expression shifts across populations (Fig. 5). Except for HARS (*P* = 0.006, *R* ^2^ = 0.38), none of the populations exhibited significant differences in gene expression between doses. Most populations showed substantial overlap in PCA space across the 0, 0.005, and 0.5 ppm treatments.

### 3.5 Inducible *versus* constitutive dose responses

Under imidacloprid exposure, several populations showed statistically significant up‐regulation of gene expression across tested doses measured in ppm: AARS (0–0.1***), HARS (0–01*, 0–10***, 0.1–10*), MD (0–10**), ME (0.1–10*, 10–100*), ND (0.1–10**), and NY (0–0.1***, 0–10***, 0.1–100***, 10–100***). For down‐regulated genes under imidacloprid exposure, only the NY population showed significant gene expression changes between doses (0–0.1*, 0–10*, 0.1–100**, 10–100***). All observed responses were non‐significant across doses (0, 0.005, 0.5 ppm) and populations for isocycloseram‐treated beetles.

## 4 DISCUSSION

Previous studies have shown that insecticide resistance in *L. decemlineata* is both phenotypically variable and genetically complex.^4^, ^13^, ^18^, ^24^, ^46^ In this study, we assessed phenotypic resistance and gene expression responses in multiple *L. decemlineata* populations following exposure to imidacloprid and isocycloseram, with a focus on metabolic detoxification as a potential genetic mechanism of cross‐resistance. Overall phenotypic evidence supported cross‐resistance across populations, but this pattern was not clearly mirrored through genetic analysis of genes linked to metabolic detoxification.

Phenotypic assays generally supported our hypothesis that *L. decemlineata* populations would exhibit greater insensitivity to imidacloprid than to isocycloseram. With the exceptions of the ND and second generation HARS populations, LC values followed reported patterns of elevated resistance to imidacloprid in the northeast and midwestern populations.^2^, ^6^, ^47^ Isocycloseram LC values were consistently lower than those of imidacloprid and showed low amounts of variance across all L. decemlineata populations. However, the significant positive correlation between imidacloprid and isocycloseram indicates an observable, pre‐existing insensitivity to the new compound and suggests potential shared resistance mechanisms even across distinct MoAs (Fig. 1).

In contrast to our hypotheses surrounding factors affecting transcript expression in response to insecticide challenges, no significant, observable patterns were supported across insecticide treatment, population, or dose. Within the control, imidacloprid, and isocycloseram treatment groups, PCA and PERMANOVA analyses showed no consistent pattern of population response arose within or across treatment groups, indicating highly variable genetic responses across populations following insecticide exposure (Fig. 2). Further examination of specific population responses to the three treatments revealed significant differences in gene expression response among treatments, but no patterns of gene expression varied according to phenotypic resistance status (Fig. 3). There were very few significant effects of dose in either insecticide treatment across populations, with the exception of ME in the imidacloprid treatment group (Fig. 4) and HARS in the isocycloseram treatment group (Fig. 5).

Examination of the differentially expressed gene sets revealed representation from multiple detoxification‐associated families. Specifically, the candidate gene list examined here includes 52 cytochrome P450s, 43 esterases, 13 glutathione S‐transferases, and 42 ABC transporters, which represent major gene families associated with detoxification in *L. decemlineata*.^14^ In imidacloprid‐treated samples, DEGs included cytochrome P450 9e2‐like (LOC111507099), glutathione S‐transferase 1‐like (LOC111509532), glutathione S‐transferase D2‐like (LOC111509814), and several multidrug resistance‐associated proteins (LOC111502194, LOC111512003, LOC111515719). In isocycloseram‐treated samples, DEGs comprised multiple cytochrome P450s (LOC111504218, LOC111508711, LOC111508880, LOC111508881, LOC111513747), glutathione S‐transferase 1 (LOC111515122), venom carboxylesterase‐6‐like (LOC111505753), esterase E4‐like (LOC111508891), liver carboxylesterase 1‐like (LOC111512274), and multidrug resistance‐associated proteins (LOC111502767, LOC111515003, LOC111517473). Although no identical loci were shared between the imidacloprid and isocycloseram DEG sets, the recurrent involvement of the same detoxification gene families indicates functional convergence on similar molecular pathways despite compound‐specific expression profiles. These findings are consistent with prior studies demonstrating that insecticide resistance in *L. decemlineata* frequently involves repeated recruitment of detoxification gene families such as CYP450s, esterases, GSTs, and ABC transporters, but with variable utilization of paralogs across populations.^22^, ^30^, ^42^, ^44^ A limitation of this study is that our transcriptomic analysis was conducted using NCBI scaffold‐level assembly (GCA_024712935.1) rather than the subsequently released Yan *et al*. chromosome‐level assembly and Wilhelm *et al*. updated version and gene expression atlas. While the mapping rates for the samples used in this study were higher with the NCBI reference, future studies would benefit from utilizing the chromosome‐level assembly to enhance genomic context and potentially improve gene annotation accuracy.^48^

Finally, consistent dose‐associated increases in transcript abundance across populations suggest that expression of this subset of metabolic detoxification genes exposed to imidacloprid are inducible, irrespective of phenotypic resistance level. These data contradict our hypothesis that populations exhibiting elevated phenotypic resistance would demonstrate constitutive gene expression across doses, whereas susceptible populations would display inducible gene expression. The results also contradict previous support for constitutive up‐regulation of genes linked to metabolic detoxification across distinct populations.^13^ The significant dose‐dependent gene expression observed specifically in ME for imidacloprid and in HARS for isocycloseram may reflect population‐specific differences in inducible detoxification responses. ME's dose‐dependent expression patterns could indicate reliance on inducible rather than constitutive detoxification systems for imidacloprid, allowing for scaled metabolic responses proportional to insecticide challenge. HARS, as a first‐generation field population from Wisconsin, exhibited the most pronounced dose‐responsive gene expression to isocycloseram, potentially reflecting regional differences in selection pressures or genetic backgrounds that influence inducible detoxification mechanisms across different compound classes. Instead, the results suggest that inducible gene expression contributes to imidacloprid resistance via metabolic detoxification, whereas isocycloseram exposure does not significantly involve the same set of inducible genetic resistance mechanisms.

## 5 CONCLUSION

Our results provide phenotypic evidence consistent with cross‐resistance in *L. decemlineata* populations for the insecticides examined; however, because only a subset of genes associated with metabolic detoxification was evaluated, no definitive conclusions can be drawn regarding the contribution of detoxification pathways to this response. Consistent evidence for phenotypic cross‐resistance across distinct and diverse populations of this beetle supports local evolution of insecticide resistance.^21^, ^22^, ^42^, ^43^ At the transcriptomic level, the absence of consistent expression patterns across treatments, populations, or doses among candidate detoxification genes suggests that resistance in *L. decemlineata* likely reflects a polygenic genetic architecture^25^, ^26^, ^27^, ^28^, ^49^ and may involve multiple resistance mechanisms beyond metabolic detoxification. Continued investigation into the molecular basis of insecticide resistance is therefore essential for improving management strategies for *L. decemlineata*, particularly in light of the cross‐resistance observed across populations in this study. Expanding our understanding of the genetic and mechanistic drivers of cross‐resistance will be critical for mitigating the evolution of resistance in agricultural systems and for developing durable, effective control strategies for this globally important pest.

## CONFLICT OF INTEREST

The authors declare no conflict of interest.

## Supporting information


**Table S1.** Location of adult *L. decemlineata* collections for imidacloprid assays.
**Table S2.** Location of adult *L. decemlineata* collections for isocycloseram assays.
**Table S3.** Candidate gene IDs, descriptions, and primer sequences.
**Table S4.** Housekeeping genes.
**Table S5.** Differentially expressed genes by treatment.
**Table S6.** PERMANOVA data.
**Table S7.** Probit regression estimates of lethal concentrations (LC_x_) for *L. decemlineata* populations exposed to Imidacloprid and Isocycloseram.
**Figure S1.** *L. decemlineata* dose response probit regression analysis to imidacloprid and isocycloseram.

## Data Availability

All data are publicly available on NCBI (PRJNA1400069) and DRYAD Dataset 10.5061/dryad.d7wm37qf3.
