# Supplementary material for: Assessing the potential for insecticide cross‐resistance to a novel insecticide in the Colorado potato beetle ( Leptinotarsa decemlineata ) using phenotypic and transcript expression analysis
Source: Pest Manag Sci. 2026 Jun 18;82(10):9908–19. doi: 10.1002/ps.71027 (PMC13569319; doi:10.1002/ps.71027)
Supplement: Supplementary file 1 [file PS-82-9908-s001.docx]

**S.Table 1.** Location of adult *L. decemlineata* collections for imidacloprid assays

| **Population** | **Location** | **Date collected** | **Assay start date** | **Beetles per rep** |
| --- | --- | --- | --- | --- |
| Lab Colony 1 | Lab susceptible, NJ, USA |  | November 22^nd^, 2019 | n = 5 x 6 reps |
| Lab Colony 2 | Lab susceptible, WI, USA |  | February 28^th^, 2020 | n = 5 x 6 reps |
| North Carolina | Camden County, North Carolina, USA | May 4th, 2021 | May 10th, 2021 | n = 5 x 4 reps |
| Maryland | Prince George County, Maryland, USA | June 2nd, 2021 | June 7th, 2021 | n = 5 x 6 reps |
| Oregon | Morrow County, Oregon, USA | May 12th, 2021 | May 17^th^, 2021 | n = 5 x 6 reps |
| Virginia | Accomack County, Virginia, USA | May 20th, 2021 | May 26^th^, 2021 | n = 5 x 6 reps |
| HARS | Waushara County, Wisconsin, USA | June 16th, 2021 | June 21st, 2021 | n = 5 x 6 reps |
| AARS | Columbia County, Wisconsin, USA | June 16th, 2021 | June 23rd, 2021 | n = 4 x 6 reps |
| North Dakota | Grand Forks, North Dakota, USA | July 28th, 2021 | August 2nd, 2021 | n = 5 x 6 reps |
| Maine | Presque Isle County, Maine, USA | June 7th, 2021 | June 14th, 2021 | n = 5 x 6 reps |
| New York | Wayne County, New York, USA | June 8th, 2021 | June 14th, 2021 | n = 5 x 6 reps |

**S. Table 2.** Location of adult *L. decemlineata* collections for isocycloseram assays

| **Population** | **Location** | **Date collected** | **Assay start date** | **Beetles per rep** |
| --- | --- | --- | --- | --- |
| Lab Colony 1 | Lab susceptible, NJ, USA |  | November 22^nd^, 2019 | n = 5 x 6 reps |
| Lab Colony 2 | Lab susceptible, WI, USA |  | February 28^th^, 2020 | n = 5 x 6 reps |
| North Carolina | Pasquotank County, North Carolina, USA | April 21st, 2020 | April 27th, 2020 | n = 4 x 6 reps |
| Maryland | Prince George County, Maryland, USA | May 15th, 2020 | May 22nd, 2020 | n = 4 x 6 reps |
| Oregon | Morrow County, Oregon, USA | May 19th, 2020 | May 25th, 2020 | n = 4 x 6 reps |
| Virginia | Accomack County, Virginia, USA | June 1st, 2020 | June 8th, 2020 | n = 4 x 6 reps |
| HARS | Waushara County, Wisconsin, USA | June 3rd, 2020 | June 8th, 2020 | n = 4 x 6 reps |
| AARS | Columbia County, Wisconsin, USA | June 12th, 2020 | June 15th, 2020 | n = 4 x 6 reps |
| North Dakota | Grand Forks, North Dakota, USA | June 23, 2020 | June 29th, 2020 | n = 4 x 6 reps |
| Maine | Aroostook County, Maine, USA | June 22rd, 2020 | June 29th, 2020 | n = 4 x 6 reps |
| New York | Wayne County, New York, USA | June 14th, 2020 | June 20th, 2020 | n = 4 x 6 reps |

**S. Table 3.** Candidate Gene IDs, Descriptions, and Primer Sequences

| **Candidate GENE ID** | **Description** | **Sequence** | **PrimerF** | **PrimerR** | **Notes** |
| --- | --- | --- | --- | --- | --- |
| **111501878** | ATP-binding cassette sub-family G member 4 | XM_023155798.1 | ACAGACGGACTTATGAGATGTTCA | GGAACTGCCGCCATGTTTTT | Spans exon-exon boundary |
| **111501880** | glutamate receptor 1-like | XM_023155796.1 | GCTTTCTGCCAACAAGGAATGA | TCGAGAGAGTGAGCATAGACA | Spans exon-exon boundary |
| **111501970** | ATP-binding cassette sub-family D member 3 (LOC111501970) | XM_023155905.1 | GGCACCAGAATTGAAAATGCCA | GGTATCGCCAAGAAGTAGGTCA | Spans exon-exon boundary |
| **111501956** | Leptinotarsa decemlineata disintegrin and metalloproteinase domain-containing protein 11 (LOC111501956) | XM_023155891.1 | GCTGGCTTGGATCAGTCTCA | GTCCCGTTGTTGTCTTGCAC | Spans exon-exon boundary |
| **111501977** | Leptinotarsa decemlineata retinol dehydrogenase (LOC111501977) | XM_023155911.1 | CATATTCGAAAATTCCATTTGTGGC | TTTGTGGGAGCCTCGACTA | Spans exon-exon boundary |
| **111502194** | multidrug resistance-associated protein 4-like (LOC111502194) | XM_023156204.1 | TGAGGTGGAAGATTTATAGGTTGA | TGTTTCCGGGGAGGTATTTACA | Spans exon-exon boundary |
| **111502226** | probable cytochrome P450 49a1 | XM_023156241.1 | GGGGAGTTATAGCGGTACATGG | TGCTACTCTGGGTTGAAGCA | Spans exon-exon boundary |
| **111502307** | glutamate receptor ionotropic, NMDA 2D-like | XM_023156371.1 | CATCGTTTCCGGCGAACAAG | TCCGTTCGGCGACCAAATAA | Spans exon-exon boundary |
| **111502371** | glutamate receptor 1 | XM_023156445.1 | AGCGCCCTAAATCTGGGGAT | TGCTCAAAGATGGCACCCAA | Spans exon-exon boundary |
| **111502417** | venom carboxylesterase-6-like (LOC111502417) | XM_023156494.1 | AAAGCCACAAGGAAGCGTTG | CTTGGTGGTACAGGGTCTTTGA | Spans exon-exon boundary |
| **111502431** | neuronal acetylcholine receptor subunit alpha-7-like | XM_023156509.1 | ACGCATCTGATTTCGCAGGT | GAATTGGGGATCAGCGTTGT | Spans exon-exon boundary |
| **111502591** | UDP-glucuronosyltransferase 1-9-like | XM_023156703.1 | AGCAAGATATCCTAGCTCATCCC | GCGAGTATTGGTACACCATGATA | Spans exon-exon boundary |
| **111502764** | probable multidrug resistance-associated protein lethal(2)03659 | XM_023156913.1 | TCATGGATGCACTGTGCTCTT | ACATTACCAGCACTTGTACCTTC | Spans exon-exon boundary |
| **111502767** | multidrug resistance-associated protein 4-like | XM_023156918.1 | ATGTGAAGACTCAATTCGAACG | TGGGCCTTCCTTTTTCTTGGT | Spans exon-exon boundary |
| **111502768** | probable multidrug resistance-associated protein lethal(2)03659 | XM_023156922.1 | GGCTACGTTTGGGTGGAGAT | CCCATGGTTCCACGATACTGT | Spans exon-exon boundary |
| **111502821** | cholinesterase-like | XM_023156986.1 | GCAGAACAATGCGACAGCTT | TTCCGGTTTGACCATCGGAG | Spans exon-exon boundary |
| **111502951** | cytochrome P450 4d2-like | XM_023157145.1 | CGTAACAAGAGACGAAGGGGA | TCTGAGGAGAGAGGGACGAC | Spans exon-exon boundary |
| **111503064** | cytochrome P450 4g15 | XM_023157289.1 | TACCGGAACGTAGTGCCAAC | TGGCGTATTTACGTCCCACA | Spans exon-exon boundary |
| **111503211** | transient-receptor-potential-like protein | XM_023157464.1 | GAGCCCAAATTGCGTCTCAAA | TGGCTTCCTCCATGGCTTTT | Spans exon-exon boundary |
| **111503224** | major facilitator superfamily domain-containing protein 6-B | XM_023157473.1 | CTTGTGTCAAGTTGCAGCTACC | CCTGTTGTTCATCAACCGTCG | Spans exon-exon boundary |
| **111503276** | voltage-dependent L-type calcium channel subunit beta-2 | XM_023162359.1 | CACTTTGCCAGCACACCAC | CCCTCGTAGTCCTCGTGTGA | Spans exon-exon boundary |
| **111503313** | para-nitrobenzyl esterase-like | XM_023157595.1 | TTCTGCCGAAGGGATATTGGG | GCTAGCCCCTTTCGAACTCTT | Spans exon-exon boundary |
| **111503433** | probable multidrug resistance-associated protein lethal(2)03659 | XM_023157722.1 | TGCATCCTTGATCAGCTCCA | CTCCGGCCTTTGTACCTCTG | Spans exon-exon boundary |
| **111503441** | cytochrome P450 4C1-like | XM_023157739.1 | AGCTCATCGACAAGGCTGAC | CGTTTCTTCCATTTGAGTCCTGT | Spans exon-exon boundary |
| **111503690** | cytochrome P450 4c3-like | XM_023158052.1 | CCCGCAATTGCATTGGACAG | GGAGTCGCTGGACGTAGTTC | Spans exon-exon boundary |
| **111503873** | sodium channel protein 60E-like | XM_023158303.1 | TCACCAGGAGGAGGAGATCG | GCGTGAGGATCGTACTTGCT | Spans exon-exon boundary |
| **111503889** | probable multidrug resistance-associated protein lethal(2)03659 (LOC111503889) | XM_023158318.1 | GGCTTTTCAATTGGCCCTGG | TGCCAGCACTGACATCTGTA | Spans exon-exon boundary |
| **111503923** | ATP-binding cassette sub-family F member 2 | XM_023158359.1 | AAATGAGCGCAGAAGAGGCT | TGGATGGACCGCTAATGAACC | Spans exon-exon boundary |
| **111503932** | diacylglycerol kinase eta-like | XM_023158372.1 | CCATCGTTACCAGTGCAACTT | ACCTTGGTTATCACCATCCACA | Spans exon-exon boundary |
| **111503968** | voltage-dependent calcium channel subunit alpha-2/delta-3-like | XM_023158421.1 | CCTCTCCACGACAATAGACGC | CACCCCCAGAGTGAAAGGAC | Spans exon-exon boundary |
| **111503985** | multidrug resistance-associated protein 7 | XM_023158438.1 | TATGACTGACTGGTGGCTGG | GATCAACCTGAAGGCCCGAA | Spans exon-exon boundary |
| **111504023** | probable multidrug resistance-associated protein lethal(2)03659 | XM_023158495.1 | AAGTGGTCGAGCAACGATGA | ACTTGCCAGAACCTACTGCG | Spans exon-exon boundary |
| **111504113** | glutathione S-transferase-like | XM_023158604.1 | GCCGGCACTAAAACCAACTAT | ATGGATGTGGACTGGTGAGC | Spans exon-exon boundary |
| **111504175** | major facilitator superfamily domain-containing protein 6-like | XM_023158674.1 | ATGTCTGCCGTTACTCCAGC | GGATGCCAACAGTACCCTGA | Spans exon-exon boundary |
| **111504198** | protein tramtrack, beta isoform | XM_023158701.1 | ACGCAAAAATCGGTTTCGGA | TCAGTGGTTAATCATTGGCTCG | Spans exon-exon boundary |
| **111504218** | cytochrome P450 4c3-like | XM_023158724.1 | CCGACGCAGTTGGAAAAAGG | TGCATACGGTTTTCCAAGACA | Spans exon-exon boundary |
| **111504278** | cytochrome P450 9e2-like | XM_023158796.1 | GGAAGCGAGAAAAAGAGGACC | GCATGCCCTGCTCCAAAGTT | Spans exon-exon boundary |
| **111504327** | voltage-dependent calcium channel subunit alpha-2/delta-3-like | XM_023158855.1 | CATGCGACACTTTCCAGCAAT | TGAACGCTTCGGTGTACCAT | Spans exon-exon boundary |
| **111504333** | ATP-binding cassette sub-family A member 3-like | XM_023158862.1 | TCATCCTCGGCAGCTTCATC | AACAGGCTTTACTCCGCACA | Within-exon |
| **111504353** | cytochrome P450 6a8-like | XM_023158887.1 | AGCGCTCACCTCTTATCCCT | CCTTTCAATTTAGCGCCCGAG | Spans exon-exon boundary |
| **111504399** | probable cytochrome P450 6a14 | XM_023158935.1 | CCGTTGGCCGGAAATTTGTT | GCATCATTTTCACTTTCCCTGA | Spans exon-exon boundary |
| **111504404** | transient receptor potential cation channel protein painless-like | XM_023158946.1 | AACAAGTGGTTCTGTGTGGG | TCCTCTTCACATTAGTCACTTCAA | Spans exon-exon boundary |
| **111504406** | conserved oligomeric Golgi complex subunit 3 | XM_023158951.1 | GCGCCCTTTCAGGTAGACTT | AATTAGGCCGACAGCAGCTT | Spans exon-exon boundary |
| **111504603** | voltage-dependent T-type calcium channel subunit alpha-1G-like | XM_023159243.1 | CATTTTTGCCGCCTTGGGT | CGTGCTCACCCAAACCTTGA | Spans exon-exon boundary |
| **111504729** | neuronal acetylcholine receptor subunit alpha-7 | XM_023159409.1 | GAATGGGATTTGCTGGGGGT | TGCGGATGATTATGACGAAGGT | Spans exon-exon boundary |
| **111504780** | UDP-N-acetylglucosamine--peptide N-acetylglucosaminyltransferase 110 kDa subunit | XM_023159470.1 | AAATCAGCCTCAAGGTCGCT | AGAAGTCTGAACTTGGCCCG | Spans exon-exon boundary |
| **111504804** | carboxylesterase 1D-like | XM_023159493.1 | ACTCTGGAAGGACGGGGAAT | TTGCCGCTCCAATAACCCAT | Spans exon-exon boundary |
| **111504808** | carboxylesterase 1D-like | XM_023159508.1 | TGGGATTACTCGAAGGGGGA | TCAAGTCAATCGTCTCCGCT | Spans exon-exon boundary |
| **111504969** | probable multidrug resistance-associated protein lethal(2)03659 | XM_023159692.1 | TGCTTTGGTCTTTGGCTGGA | CCTCCGTGGATAGCTTCACC | Spans exon-exon boundary |
| **111504976** | methyl farnesoate epoxidase-like | XM_023159708.1 | GAAGACTTTGACGGACGACCA | TCGGAGAACACCAAACCTAACC | Spans exon-exon boundary |
| **111504977** | probable cytochrome P450 305a1 | XM_023159709.1 | AGAATTTTCCGCCAGGTCCA | TTGCCCTCCTAAAGCTGCTG | Spans exon-exon boundary |
| **111505109** | ribosome biogenesis regulatory protein homolog | XM_023159865.1 | AGATACTGAACTTCTTAGGAAACGA | TGATTAACCAGCAACTGGGCA | Spans exon-exon boundary |
| **111505188** | voltage-dependent T-type calcium channel subunit alpha-1G-like | XM_023159960.1 | CCCACATACCTATGCCAACTGAA | GCATGCCCGAGTTGTTGTTT | Spans exon-exon boundary |
| **111505245** | ionotropic receptor 93a-like | XM_023160021.1 | GGGGCTACGATGATTGACGA | GCCATGGGGGATTATGAAAGG | Spans exon-exon boundary |
| **111505305** | cytochrome P450 4d2-like | XM_023160082.1 | GATGCTCTGTTGCAGTTGCC | CCAAGGTATCGTTTCCCCCA | Spans exon-exon boundary |
| **111505381** | transient receptor potential channel pyrexia | XM_023160177.1 | AGATGAGAATGGCAGGAGCG | GTTGGCACCATGACCAAGGA | Spans exon-exon boundary |
| **111505524** | cytochrome P450 6k1-like | XM_023160352.1 | CGTTTGTAGTACAGATTCAGCAAGT | AAGGCTATGGTGGAACTGGT | Spans exon-exon boundary |
| **111505535** | acetylcholine receptor subunit alpha-like | XM_023160363.1 | TCCTCCGGAGATACACAAGTCT | CCAGTCCTCCTTCACCTTCG | Spans exon-exon boundary |
| **111505741** | venom carboxylesterase-6-like | XM_023160598.1 | ATATTGCTCGCTTGTTTTGCGA | ACAGGGTCCCCCTTATACTGC | Spans exon-exon boundary |
| **111505753** | venom carboxylesterase-6-like | XM_023160617.1 | ACACACAGTGAAAGGACGTT | CTGGAGGGACTGATTCCTGG | Spans exon-exon boundary |
| **111505902** | cytochrome P450 CYP12A2-like | XM_023160796.1 | TTATTCTTACCAGGCGGGGC | ATTGGCCCAAGTTGCTTACTG | Spans exon-exon boundary |
| **111505971** | ATP-binding cassette sub-family B member 8, mitochondrial-like | XM_023160887.1 | AGTATTGATTCAGCCGAGGATT | TCATGAATAAACGATTGTGAGTGAA | Spans exon-exon boundary |
| **111505998** | cholinesterase-like | XM_023160912.1 | ATTGCGATGAGTGGAAAGAGC | CGCAGGCAAAGGTAGTTGCT | Spans exon-exon boundary |
| **111506177** | ATP-binding cassette sub-family B member 6, mitochondrial | XM_023161180.1 | GTCAAAAGGGGAGCTGTGGA | TCCCTGGCGGTACAGAAAAC | Within-exon |
| **111506182** | venom carboxylesterase-6-like | XM_023161186.1 | TCCAGATGTTCCACCTTCCG | GGCGATGGGTTTCCGGTTTT | Spans exon-exon boundary |
| **111506296** | transient receptor potential cation channel trpm | XM_023161323.1 | AACACGCTTCGCAGATCAAC | GCTGAAGAACCTGAAGAACCATT | Spans exon-exon boundary |
| **111506313** | glutathione S-transferase theta-1-like | XM_023161347.1 | GGCGATGGCCCATTTGTATTT | ATCCTACCATTCGTGGCTGT | Spans exon-exon boundary |
| **111506321** | acetylcholine receptor subunit beta-like 1 | XM_023161356.1 | CCAAAGCTACGGAAGCGGTA | TCCAGTCTTCACGGGTCTGT | Spans exon-exon boundary |
| **111506324** | muscle calcium channel subunit alpha-1-like | XM_023161359.1 | GGTGATGCCACCTTTGGGAA | TCGATAACGATCGCCAATACTTAG | Spans exon-exon boundary |
| **111506564** | acetylcholine receptor subunit alpha-like 1 | XM_023161680.1 | TTGGGTTGAGCAGGAATGGA | TCAGAGGGAACATGGAGGGT | Spans exon-exon boundary |
| **111506689** | probable cytochrome P450 6a13 | XM_023161849.1 | TTTAATGGTCGGGTCTGCCA | ATCTTTTTGTCAGCAGCGTG | Spans exon-exon boundary |
| **111506755** | cytochrome P450 18a1 | XM_023161958.1 | CGTGAATGACGTTTCTTTGGCA | TGTTGGATCTGACGATCGGG | Spans exon-exon boundary |
| **111506798** | BLOC-1-related complex subunit | XM_023162001.1 | TGTTGGATCTGACGATCGGG | CGTCTTCCTCCAGATCAGCC | Spans exon-exon boundary |
| **111506979** | glutamate [NMDA] receptor subunit 1-like | XM_023162201.1 | TCGTTTGGGAAGAGATCGGC | CTATTTCCCGGCCACACGAT | Spans exon-exon boundary |
| **111507074** | venom carboxylesterase-6-like | XM_023162320.1 | TCCAGAGAAGTGAGTAGGGCT | TGCATCGGTGTAGAGCTTCG | Spans exon-exon boundary |
| **111507083** | glutamate receptor ionotropic, kainate 1-like | XM_023162330.1 | TGTTCTTCAAGAGGACGGACT | TGTTCTTCAAGAGGACGGACT | Spans exon-exon boundary |
| **111507098** | cytochrome P450 9e2-like | XM_023162345.1 | ACAGTTTAGTGGTTGTGATAGTTTT | AAAATAATACAGGGCGGCCA | Spans exon-exon boundary |
| **111507099** | cytochrome P450 9e2-like | XM_023162347.1 | GAGACGGCAGAGTGTCCTAC | AACCCGGTGGGTATCTTCTG | Spans exon-exon boundary |
| **111507106** | cytochrome P450 9e2-like | XM_023162355.1 | CCTGTGGATGAATAAAGCTTTCAGA | TAAATGAAAAGGAGAACCACAGTGA | Spans exon-exon boundary |
| **111507163** | voltage-dependent calcium channel subunit alpha-2/delta-3-like | XM_023162430.1 | TCTCAACTTCATTTCAGTAGCCT | TGCCCATTGTGAAACCTCTTC | Spans exon-exon boundary |
| **111507260** | venom carboxylesterase-6-like | XM_023162548.1 | AGGCAGGTACAATAAGGTACTTCAG | TGAATTTGGATTGCAGTTCAGC | Spans exon-exon boundary |
| **111507261** | cholinesterase-like | XM_023162551.1 | ACCATATTTGGGCAAAGTGCAG | ACAGGATTACAGTGCCTTTGGA | Spans exon-exon boundary |
| **111507301** | venom carboxylesterase-6-like | XM_023162594.1 | AGTCCAGGTTCTGCAGGTCT | CTTGTCGATCGTGAGCCCAA | Spans exon-exon boundary |
| **111507303** | venom carboxylesterase-6-like | XM_023162595.1 | CACTGGGAAAACAACAGCCA | GCTGGCTGAGGATCCTTGAA | Spans exon-exon boundary |
| **111507304** | venom carboxylesterase-6-like | XM_023162596.1 | CCAGGTGCAGAAACAGGTCT | CGGAACTGCCAGGTTTCACT | Spans exon-exon boundary |
| **111507306** | venom carboxylesterase-6-like | XM_023162599.1 | TGTTTACACCACGGTGGGAC | TGCATGACCGTGAACGAATG | Spans exon-exon boundary |
| **111507339** | chloride channel protein 2-like | XM_023162635.1 | TCAAATCTCGAATCGCCCAGT | TTCAGCTGTTCGCTTTCCCA | Spans exon-exon boundary |
| **111507340** | chloride channel protein 2-like | XM_023162636.1 | GACCACGGAAATGCTTGCTG | TGCTGAACAGTACCCCTCCA | Spans exon-exon boundary |
| **111507343** | chloride channel protein 2 | XM_023162638.1 | GAACTGGACAAGCCCGTGTA | CGTGAATCCCCTGAGCGTAA | Spans exon-exon boundary |
| **111507523** | antichymotrypsin-2-like | XM_023162841.1 | GCAGCTGATAACGAGGCTCT | TTCACACTTTCTGCTTGTTTCTTA | Spans exon-exon boundary |
| **111507644** | ATP-binding cassette sub-family D member 2 | XM_023162987.1 | ACGTTTGGTCAGGAACAGGTAT | TTTTTCGGTACTCTGCGGGT | Spans exon-exon boundary |
| **111507680** | venom carboxylesterase-6-like | XM_023163026.1 | AGGTCGTGATAGCGGGTACT | AGGCTTTGAAATGAAAGCATCGT | Spans exon-exon boundary |
| **111507684** | major facilitator superfamily domain-containing protein 6 | XM_023163033.1 | ACAGTGTTTGGCAGGAGAGG | ACATCGTGTGGATGTGACCC | Within-exon |
| **111507687** | venom carboxylesterase-6-like | XM_023163036.1 | TTCCCTGAGATTTAGGGACCCA | TGAACTGTGTCTCTACGCACC | Spans exon-exon boundary |
| **111507722** | venom carboxylesterase-6-like | XM_023163068.1 | TTGAGGTACTCCGCAAGGTG | CTGCTTGGGGTATGGACACA | Spans exon-exon boundary |
| **111507800** | venom carboxylesterase-6-like | XM_023163157.1 | TTTACGATTCGAGCCGCCTG | CTTCCTTGGGTGCATACTGGT | Spans exon-exon boundary |
| **111507849** | multidrug resistance-associated protein 4-like | XM_023163218.1 | ACTTCTGGGATACACGATTGGT | TTTGCTCCAAGTTGACCCATA | Spans exon-exon boundary |
| **111507901** | muscle calcium channel subunit alpha-1-like | XM_023163284.1 | TCGGAAGGAATGTTTCGGCA | GTTCATGCTGCAGCCCTCAT | Spans exon-exon boundary |
| **111507913** | cytochrome P450 9e2-like | XM_023163302.1 | TGATCACTTCACTGACCACAGA | TCTCGCCATTTTCTACCTCTCA | Spans exon-exon boundary |
| **111507992** | juvenile hormone esterase-like | XM_023163398.1 | AGAACGAATCTAAATGGGGGTGA | CGCAGGAAGTGGAGCCATAA | Spans exon-exon boundary |
| **111508023** | soluble guanylate cyclase 89Db-like | XM_023163440.1 | CGACATCGACTCACACGGTT | TGTTGCAGCTGACCCATGAT | Spans exon-exon boundary |
| **111508040** | ATP-binding cassette sub-family G member 4-like | XM_023163459.1 | GAGAAGACTCGCAGTTGCCT | CCACATTATCCAAACCCGTGG | Spans exon-exon boundary |
| **111508081** | glutamate receptor-interacting protein 1 | XM_023163506.1 | CAGGTCTAACCGCAATCGGA | TTCGTGAAAGAGCTGGCACC | Spans exon-exon boundary |
| **111508105** | probable cytochrome P450 303a1 | XM_023163528.1 | GCTGTTTCACTGTTCCGCAC | AATGACCAAAACGTCCTTCGG | Spans exon-exon boundary |
| **111508106** | methyl farnesoate epoxidase-like | XM_023163529.1 | GGCCCATCTCAAAATCGAAACC | GGCCCATCTCAAAATCGAAACC | Spans exon-exon boundary |
| **111508293** | glutamate receptor ionotropic, kainate 2-like | XM_023163739.1 | GGGCCCCACCATAGAAAGTG | ACGTCGAACCACCTCCTAGA | Spans exon-exon boundary |
| **111508363** | transient receptor potential cation channel protein painless-like | XM_023163833.1 | TGATGTGGAATACAAGGAACTTGA | CAGGTAGTCAATACACCTGCTCA | Spans exon-exon boundary |
| **111508437** | para-nitrobenzyl esterase-like | XM_023163930.1 | CCTGGTGCTTTCGTGAAGTATT | CTGGGGAAAACTTTGAAGCCAG | Spans exon-exon boundary |
| **111508632** | probable cytochrome P450 301a1, mitochondrial | XM_023164201.1 | CTTGAAGGGCTTTTTGGGGC | TCCATGCAGGGGTGTTGAAA | Within-exon |
| **111508711** | cytochrome P450 315a1, mitochondrial | XM_023164304.1 | TCGGACCCGTTTCTTGTGTT | TGTGAACCGGATATTTTCCTTCT | Spans exon-exon boundary |
| **111508869** | transient receptor potential-gamma protein-like | XM_023164496.1 | CGGCTTCCAGGAATACTAGCC | CCGTTTCAAAGCCTTCACGG | Spans exon-exon boundary |
| **111508872** | cytochrome P450 9e2-like | XM_023164498.1 | GATCAGTTGGCAGTGTTGTCG | TTGCAGCATCTGATACAATCTATTT | Spans exon-exon boundary |
| **111508879** | cytochrome P450 3A19-like | XM_023164504.1 | GCCGAGGAGTTGTATAGGTCA | GCGCTGACAATCAGCTTGAG | Spans exon-exon boundary |
| **111508880** | cytochrome P450 6a2-like | XM_023164506.1 | AGGAGTTGTGTAGGACTTCGT | TTGGTTGTAGGGCTGACAGA | Spans exon-exon boundary |
| **111508881** | cytochrome P450 6a2-like | XM_023164508.1 | TGGTCCAAGAAGTTGTATAGGTCT | ATGGGCTCACAGCGATTCTG | Spans exon-exon boundary |
| **111508891** | esterase E4-like | XM_023164524.1 | GCCCAAAGTTACACCAAACGA | GCGTTGCTTCCAGGCTTTG | Within-exon |
| **111508919** | cytochrome P450 9e2-like | XM_023164562.1 | TGAGACGTCAAAGTACCGCC | TTGATACAGTCCTGAGTACCTCG | Spans exon-exon boundary |
| **111508973** | voltage-dependent T-type calcium channel subunit alpha-1G-like | XM_023164631.1 | TAGCAGACTGCATCGGAGGG | TCCTGACTCCGACTCTTCCAT | Spans exon-exon boundary |
| **111509140** | pyrethroid hydrolase Ces2a-like | XM_023164831.1 | CGTTACCACGTGCAATATGAGG | TCGTCGTCCAGTTTTCCAGT | Spans exon-exon boundary |
| **111509240** | cytochrome P450 6k1-like | XM_023164943.1 | GACTGACTGCGGGGGTATTT | ACTCACAAAGGGCAACCCTAA | Spans exon-exon boundary |
| **111509258** | glutamate receptor ionotropic, kainate 2-like | XM_023164970.1 | GCCCTTCATGACTTTAGGCGT | GCCGTGGCAAAGGGATCTAA | Spans exon-exon boundary |
| **111509288** | collagen alpha-2(IV) chain | XM_023165000.1 | TGTATGCCAACCAGGACGAC | CGCTCCCGGTAAACCTTGAT | Spans exon-exon boundary |
| **111509350** | probable cytochrome P450 6a14 | XM_023165075.1 | GCAGACCAGACTTTTTGCAGC | ACCAATGGGGAGTTTCACCT | Spans exon-exon boundary |
| **111509378** | transient receptor potential channel pyrexia-like | XM_023165106.1 | TCGCGGAAATAGGTGGAAGC | CGTCCTTCCTTCCTCATCGG | Spans exon-exon boundary |
| **111509471** | glutathione S-transferase 1-like | XM_023165231.1 | CCTTTGGGAAAGCAGGGCTA | ACCAGAGCTCTCTTCTTGGGA | Within-exon |
| **111509532** | glutathione S-transferase 1-like | XM_023165296.1 | AGAGCTGTCGGAGTGGACTT | ACAGTGCTGAGGGTTGATCTTA | Spans exon-exon boundary |
| **111509814** | glutathione S-transferase D2-like | XM_023165644.1 | GGACGGAGTTAAAGGAGCAGAA | ATGAGCCGCATGCCAAGT | Spans exon-exon boundary |
| **111509857** | pickpocket protein 11-like | XM_023165700.1 | CCCAACGGTCAGAAAATGGA | GGGCGTGGAAAAATACCCTG | Spans exon-exon boundary |
| **111509916** | venom carboxylesterase-6-like | XM_023165775.1 | AGGATCTATTCCACAGGGGCT | CCGACCACTTCAGCCTTCTT | Within-exon |
| **111509981** | cytochrome P450 4C1-like | XM_023165844.1 | GCGTGTGAAAGGGAGTTGTT | GACGGCGGGGGATTTGAAAG | Spans exon-exon boundary |
| **111510069** | multidrug resistance protein 1B-like | XM_023165927.1 | AATTGTGCCTTGCCGGGATA | TTTGGGCGAACCTGGTAGTC | Within-exon |
| **111510079** | chloride intracellular channel exc-4 | XM_023165939.1 | CGATGGCTGATGAAGTTTCAGA | TCGATTGTCGATGCCCTTATGA | Spans exon-exon boundary |
| **111510198** | multidrug resistance protein homolog 49-like | XM_023166095.1 | TGCTACTGTTTTCAAAGTCTGCG | CGCAACAAGACCCTTCTGGA | Spans exon-exon boundary |
| **111510322** | major facilitator superfamily domain-containing protein 12-like | XM_023166225.1 | CAACGTGGGATTTCCAAATTCTT | GCGGGATGAGTCATAGGTTCA | Spans exon-exon boundary |
| **111510344** | ATP-binding cassette sub-family A member 5-like | XM_023166246.1 | TACCAGGGGTCGAGTCCAAA | ATAAGGCATCGTGTTGGGGG | Spans exon-exon boundary |
| **111510478** | ATP-binding cassette sub-family E member 1 | XM_023166409.1 | TGGCTGGCAATTTAGAAGCTG | AGGATATTTGTTGAGATCGACTTGT | Spans exon-exon boundary |
| **111510577** | major facilitator superfamily domain-containing protein 10 | XM_023166498.1 | ACCTGCTTTATTCGCCCTGT | GGCACGGTTTTCCTTGGGTA | Spans exon-exon boundary |
| **111510654** | cytochrome P450 6k1-like | XM_023166585.1 | GCCGTCGACAGGTTTAGTCA | GAATTTCTCGGGTTCCGGGT | Within-exon |
| **111510681** | splicing factor 1 | XM_023166613.1 | ACAAGTCTTGGCTGTGTCCA | TGGACCTACAATCACGAGCG | Spans exon-exon boundary |
| **111510688** | probable cytochrome P450 6a23 | XM_023166624.1 | AGAACAGAGCTAGAAGACACCC | TCCAAATCTGGCCCCTATGC | Spans exon-exon boundary |
| **111510689** | probable cytochrome P450 6a23 | XM_023166625.1 | ACCGACCTTCACATCAGGAAA | TGAATGACCGAGTATTCATTGAGGA | Spans exon-exon boundary |
| **111510741** | esterase FE4-like | XM_023166679.1 | GCTGCAGCTCAGATCCCATT | GGCAAGTTGCTGCTTTCGAT | Spans exon-exon boundary |
| **111510744** | esterase B1-like | XM_023166682.1 | TCACCGATGGCAAAAGGTCT | TTGCTGCAACTTGCCCAAAC | Spans exon-exon boundary |
| **111510839** | cytochrome P450 6k1-like | XM_023166792.1 | ACTACCAGAAGAATTAGCAACCA | TCGAAGAGCTGGTAGTTTCG | Spans exon-exon boundary |
| **111510879** | cytochrome P450 6k1-like | XM_023166832.1 | TTGAAGATTGTGCATCGGGC | GGAAGAGGATGGAAACATCTTATCG | Spans exon-exon boundary |
| **111510927** | transient receptor potential-gamma protein-like | XM_023166902.1 | GGACCGTTCTCATCCCATGAA | TCATTCTCAGCAGGACTTTGTAA | Spans exon-exon boundary |
| **111510953** | UDP-glucuronosyltransferase 1-5-like | XM_023166929.1 | AGCTTGCCAGTCTTCTTGGT | CGTAGCTCTTTGGCTCCTCT | Within-exon |
| **111511127** | voltage-dependent calcium channel type A subunit alpha-1 | XM_023167144.1 | GATTTGGAGGGTCGTCGTGT | GGCGAGTCGTGGTACTTCAT | Spans exon-exon boundary |
| **111511263** | esterase FE4-like | XM_023167276.1 | ACAAGAATTATCATGGCGGAACG | CACAGGGTACAGGAGCTTTGA | Spans exon-exon boundary |
| **111511268** | cytochrome P450 307a1-like | XM_023167286.1 | CAGAATAGTTCAATAGGAGGCTACA | ATCGGACATGCTCAGGTCAT | Spans exon-exon boundary |
| **111511368** | probable cytochrome P450 4aa1 | XM_023167379.1 | AATTACCTGGTCCAAAAGCGT | ACCCAGATGCTCCATCTCTCT | Spans exon-exon boundary |
| **111511395** | cytochrome P450 9e2-like | XM_023167410.1 | ACCACAGGGTTCTCATACACG | TTGCCATTCTTCTCCTTTGAGG | Spans exon-exon boundary |
| **111511486** | protein unc-13 homolog 4B | XM_023167501.1 | ATCCCACTCAGCACTGTTCC | CTCCCTGGCGAGTGACTTTA | Spans exon-exon boundary |
| **111511545** | cytochrome P450 6a8-like | XM_023167571.1 | AGATGCAAGATCGGAGGTGT | CACTTGTTAAAATGTCTTTGGCGA | Spans exon-exon boundary |
| **111511639** | acetylcholine receptor subunit alpha-L1 | XM_023167671.1 | CGCTGGAACACTGAGTGAGA | TTGTTCCCGAGTCGCTTTCA | Within-exon |
| **111511649** | venom carboxylesterase-6-like | XM_023167681.1 | CCTATGGCGATGACCGAACA | TGAGGAAGGATACCGTGGGG | Spans exon-exon boundary |
| **111511697** | major facilitator superfamily domain-containing protein 8 | XM_023167734.1 | GATCGAAGCCTCAGGGAGTG | ACGTTACGAAAACTGGCCCC | Spans exon-exon boundary |
| **111511764** | voltage-dependent calcium channel subunit alpha-2/delta-3 | XM_023167793.1 | AGACGTCCCTTGTCGTTGTC | TGCCCAGTTTTTCACCTCGT | Spans exon-exon boundary |
| **111511812** | ATP-binding cassette sub-family A member 3-like | XM_023167829.1 | CCTGCAGAGCTACGAGCAAT | TCTTGGCCCAGCAGTTTGAT | Within-exon |
| **111511982** | probable multidrug resistance-associated protein lethal(2)03659 | XM_023168084.1 | TGGTTAGAATTGCATTGCGTCA | GCCACCAAATGTTTCGCCTT | Spans exon-exon boundary |
| **111512003** | multidrug resistance-associated protein 4-like | XM_023168093.1 | TTTGGTCACCCACACTTGCT | TCTCGTAAATTCGCGGCCAT | Within-exon |
| **111512024** | multidrug resistance-associated protein 4-like | XM_023168112.1 | TAGAGCGCTGTGGAATTGCT | GCTTCTGGTTCCAAATAATCTCCC | Spans exon-exon boundary |
| **111512033** | neuronal acetylcholine receptor subunit alpha-7-like | XM_023168124.1 | GTCCACGTGAGAAGGTCCTG | ATTTGCTGCGGTTTCGGTTC | Within-exon |
| **111512040** | pickpocket protein 28-like | XM_023168132.1 | CGGAAGATGGCATGTGTTTCA | TTCACCCTCCATGAACACCTTT | Spans exon-exon boundary |
| **111512041** | venom carboxylesterase-6-like | XM_023168133.1 | ACCAGCCAGCTCTTGCATTA | GGCACTGTTTCAAGGAGGTTC | Spans exon-exon boundary |
| **111512151** | probable cytochrome P450 6a23 | XM_023168254.1 | AAAACAAAGCAGCAAGACATCCC | TCCATATCTGGCTCCTATGCAC | Spans exon-exon boundary |
| **111512152** | probable cytochrome P450 6a20 | XM_023168255.1 | TGTTCACAGGATTCACAATGGAC | AGAGAAAAGCCAGATCGCTAGT | Within-exon |
| **111512159** | cytochrome P450 9e2-like | XM_023168261.1 | TCACGGATCGCAGGAAGTTT | TCTCCATTTATCCCCTGTGAGT | Spans exon-exon boundary |
| **111512244** | para-nitrobenzyl esterase-like | XM_023168338.1 | TCCAGAAGAAGATGGGTTGACA | TCCGGTGTTGGATTTAGATACTT | Spans exon-exon boundary |
| **111512274** | liver carboxylesterase 1-like | XM_023168378.1 | ACTTTGCGAAAACGGGGAATC | TGCTGTCTGAAACTGTTGGC | Spans exon-exon boundary |
| **111512328** | juvenile hormone esterase-like | XM_023168438.1 | GAACTGGGGTTCGCTGGTTA | GGGATGTGATGGCAGCATGA | Within-exon |
| **111512344** | sodium channel protein Nach-like | XM_023168452.1 | CCGATGGGGTCTCCTTCATC | TGTTCCTCCAAGAGACACAATCA | Spans exon-exon boundary |
| **111512347** | cytochrome P450 4C1-like | XM_023168455.1 | ATGCTGCTGGATCCTTCCAT | GTGGTTTCACTGCCGGCTAA | Spans exon-exon boundary |
| **111512369** | cytochrome P450 307a1-like | XM_023168477.1 | AAGGAAAGGACTTCGGGGAC | TCGCAGAGGGCTAGAGAGTT | Spans exon-exon boundary |
| **111512424** | juvenile hormone esterase-like | XM_023168544.1 | TCAAATCAAACAGATCAGAAGACCT | TGTCATTCGGGACATCAGCC | Spans exon-exon boundary |
| **111512448** | UDP-N-acetylglucosamine transferase subunit ALG13 homolog | XM_023168567.1 | TTATAAGCCACGCTGGTGCT | AGTCTTCATTCACAACCACGA | Within-exon |
| **111512481** | transient receptor potential channel pyrexia-like | XM_023168612.1 | TTTCTGGTTCTTTTCCACAAATCA | TTGCAAAACGTTCTACACAAGACT | Within-exon |
| **111512494** | probable multidrug resistance-associated protein lethal(2)03659 | XM_023168631.1 | CCATTGTTGCATTGTTATGGAGA | TATGGCTGGATGACGCTTCG | Spans exon-exon boundary |
| **111512583** | multidrug resistance-associated protein 4-like | XM_023168731 | ATACCGCCAGGAAATATCGAG | TCGGTAACTATGGTTTCATCAGCA | Spans exon-exon boundary |
| **111512598** | probable multidrug resistance-associated protein lethal(2)03659 | XM_023168742.1 | CAACGCTGCAAGAAGGAAAGA | ACCCAGATTGGAGGTGAGGA | Spans exon-exon boundary |
| **111512607** | probable multidrug resistance-associated protein lethal(2)03659 | XM_023168752.1 | AGGCTGGAAGCATCAACTCG | TCTGCCTTCGAAGCTCTGAT | Spans exon-exon boundary |
| **111512633** | multidrug resistance-associated protein 4-like | XM_023168778.1 | TCTGGGTTTTTATCTGAGAGCCA | CCCATCCAAATGTGGGCAGT | Within-exon |
| **111512651** | acetylcholine receptor subunit alpha-like | XM_023168796.1 | AGCTGGACAGCAGAAGTCAC | ATTGGCGGTTCTCCCAAGTC | Spans exon-exon boundary |
| **111512692** | E3 ubiquitin-protein ligase SINAT4-like | XM_023168838.1 | CGTCCCATAGAGGACTCCAGA | CAGCGATAAACAAACCGGGC | Spans exon-exon boundary |
| **111512862** | glutamate receptor ionotropic, kainate 2-like | XM_023169028.1 | AGACAAGATGAACGGGGCTT | CCAGGTCAGCTTTGTGCTCT | Spans exon-exon boundary |
| **111512865** | glutamate receptor ionotropic, kainate 2-like | XM_023169031.1 | TGATGCTCATACAAAGGCTCA | TGGTTTCATGATGTGAACCCCT | Spans exon-exon boundary |
| **111512874** | transient receptor potential cation channel subfamily A member 1 | XM_023169037.1 | ACTGTGTATCAGGTCCGGTG | CTTGGGCACATGCAAGATGA | Spans exon-exon boundary |
| **111512897** | probable cytochrome P450 6a13 | XM_023169053.1 | CAGAAGTTGACATTGCAGCCC | TCAGGAAACTCATAACCCTGGA | Spans exon-exon boundary |
| **111512969** | venom carboxylesterase-6-like | XM_023169132.1 | GCATTGACTTCTACGCCTTCC | GTGTTTCGGTGGCTGGAATC | Spans exon-exon boundary |
| **111512970** | acetylcholinesterase 1-like | XM_023169134.1 | CTGAGGAGGACAGATTAACGCT | GCGATGGTGTTGGATTTTGAT | Spans exon-exon boundary |
| **111513050** | ATP-binding cassette sub-family B member 7, mitochondrial-like | XM_023169211.1 | AACTTGGCCGATGGGATACG | AAATGGCTCTGGCTATGGCT | Spans exon-exon boundary |
| **111513254** | glutathione S-transferase C-terminal domain-containing protein homolog | XM_023169435.1 | GCCAAACCTTCAGAACCCCT | CTGGCCACTCAGCAAACTGT | Within-exon |
| **111513291** | para-nitrobenzyl esterase-like | XM_023169486 | CGACCAGCCGGTTATTCCAT | ACCATATCGCTAATGAGGTTCACA | Spans exon-exon boundary |
| **111513480** | voltage-dependent calcium channel subunit alpha-2/delta-3-like | XM_023169700.1 | ACAGTGGACAAAGCGGAGAAT | GCTGTAGGACGGCACTCTTG | Spans exon-exon boundary |
| **111513709** | esterase E4-like | XM_023169935.1 | CTTCGGTTCGAGCCTCCTGT | TGAATGCATTGGCTTTTGTCGT | Spans exon-exon boundary |
| **111513747** | cytochrome P450 6k1-like | XM_023169972.1 | AGATGTGTATCAACGAAACCCTG | AAGTCTGTCCCTGGCACTTTA | Spans exon-exon boundary |
| **111513754** | esterase B1-like | XM_023169980.1 | GGAGGACTACCATGGTGGAAA | GCTGGTACAGGAGCCTTGAA | Spans exon-exon boundary |
| **111513755** | acetylcholine receptor subunit beta-like 2 | XM_023169981.1 | AGCTCACTCTTCCATGACAGG | CTTACTCCTTGGAATGCTGCC | Spans exon-exon boundary |
| **111513767** | voltage-dependent T-type calcium channel subunit alpha-1G-like | XM_023169994.1 | ACCAATTAGGAAGCCATCCCC | CATCAAGGACCGTACTCGGA | Spans exon-exon boundary |
| **111513855** | glutathione S-transferase-like | XM_023170075.1 | GAACACGTTCCCTTCTTTCTCA | ATCAGCCCACGAAATCCTGT | Spans exon-exon boundary |
| **111513883** | venom carboxylesterase-6-like | XM_023170104.1 | GTGCGAGCGCTCTATACCAA | AAGCTCACCGCTCATCTCTC | Within-exon |
| **111514056** | glutathione S-transferase D1-like | XM_023170297.1 | GTTGCGCATTACACGTTGGG | CGTGGAAATTATCCAGGGCAA | Spans exon-exon boundary |
| **111514157** | glutamate receptor ionotropic, kainate 2-like | XM_023170394.1 | CCGTATTGACTGTAGTGGCGA | TGGTTGGAGGTCGCGTTTT | Spans exon-exon boundary |
| **111514255** | voltage-dependent calcium channel type A subunit alpha-1-like | XM_023170493.1 | AGTCTCTGGCATACCTAGTCTACA | ACGATGGCGAATAAAACCAGT | Spans exon-exon boundary |
| **111514427** | transient receptor potential cation channel subfamily V member 5 | XM_023170669.1 | CCACGACACAAACTGCTGC | TCGGTCACGTCAGGAAAATCA | Spans exon-exon boundary |
| **111514530** | venom carboxylesterase-6-like | XM_023170785.1 | CGGAGGCGGATGTTCTTACA | TGTTGGTGTCGGGTTCATGT | Spans exon-exon boundary |
| **111514589** | probable cytochrome P450 12a5, mitochondrial | XM_023170848.1 | TCACCACAAAAATCGAATAGCCA | TCCTCAGCAAGAAGGATCGC | Within-exon |
| **111514596** | voltage-dependent calcium channel subunit alpha-2/delta-3-like | XM_023170856.1 | GATCTGCGGCCTGTGTATCA | GTTTCGAGGTTCGGAGTCGT | Spans exon-exon boundary |
| **111514668** | glutamate receptor ionotropic, kainate 3-like | XM_023170919.1 | GACTGTGATGGGGTAGACAGT | AGTGAGACCTTTGTATGTTCCTGAT | Spans exon-exon boundary |
| **111514670** | cytochrome P450 4C1-like | XM_023170921.1 | AAGGTTATTTCCAGTAGCTGGACTG | CAGACTGGAAAACAAAATCACCAAC | Spans exon-exon boundary |
| **111514671** | cytochrome P450 4C1-like | XM_023170922.1 | TCCAACGTGTTTGAGGAAGG | TCTTCCATATATCAACGGGTGC | Spans exon-exon boundary |
| **111514672** | alpha-tocopherol transfer protein-like | XM_023170923.1 | CCAGAAGTTCCGAGTGACCA | TGGTGTAGTACATGTCCAGTTTCT | Spans exon-exon boundary |
| **111514902** | multidrug resistance-associated protein 4-like | XM_023171144.1 | CGTTTGGGTTCTTTTCGGATGT | CCACTATGAATGTTTTGCTTACCA | Spans exon-exon boundary |
| **111514941** | cytochrome P450 6k1-like | XM_023171184.1 | TGAAAGTGGGAGATTTTGCGG | TGCTTCGTTCAGGGCTGATT | Spans exon-exon boundary |
| **111514944** | glutamate receptor 1-like | XM_023171187.1 | AAAGTGTGGATTGCGTCCATT | TTCTTCTTCCGCTCTGGCATT | Spans exon-exon boundary |
| **111514950** | ATP-binding cassette sub-family G member 4 | XM_023171221.1 | ACGTTCAGGGGAGTTGTGTG | CCACTCATTCCTTCCGTCTTGT | Spans exon-exon boundary |
| **111515003** | probable multidrug resistance-associated protein lethal(2)03659 | XM_023171236.1 | CGAAGCAGCACATAGTCAGTG | GCGGACGAACTGAAAACAAA | Spans exon-exon boundary |
| **111515122** | glutathione S-transferase 1 | XM_023171357.1 | TCTGAATACACGTTGGCACC | ATCCAGGGCAATATGCGTTT | Spans exon-exon boundary |
| **111515157** | sodium channel protein Nach-like | XM_023171390.1 | AACTCCCACCTTCGACGATAC | TGAATGGTCCCACTCTCTTGG | Spans exon-exon boundary |
| **111515176** | glutathione S-transferase D7-like | XM_023171420.1 | TATGATTTGTGCAAGAACTTTGGG | GGTTTGGACTCACCGCCAA | Spans exon-exon boundary |
| **111515202** | glutathione S-transferase 1-like | XM_023171435.1 | AGGCTTTGGGGGTCGAATTG | TGTTGCGGATTCAGCTTCAGA | Spans exon-exon boundary |
| **111515214** | glutathione S-transferase 1-like | XM_023171444.1 | CCGACTTTCTTGCACTTAATCC | TTGCATGGCTGTCCGATAAG | Spans exon-exon boundary |
| **111515223** | glutathione S-transferase 1-like | XM_023171454.1 | AGCTTTGGGCTTGAACCTGA | GTGTTGCGGATTGAGTGCAA | Spans exon-exon boundary |
| **111515228** | multidrug resistance-associated protein 4-like | XM_023171456.1 | TGAATTCGATGAACATCAGGACG | TATGCACACCAGATCGAACCAA | Spans exon-exon boundary |
| **111515375** | glutathione S-transferase 1-like | XM_023171602.1 | TCGCTAGGATAACGGAGGTGTA | AAGTCTGCCAGGGAAAGCTC | Spans exon-exon boundary |
| **111515377** | glutathione S-transferase D5-like | XM_023171605.1 | GAAGGCGCGAAAACAGTGAG | CAGCTGGGCTGACGAAATTA | Spans exon-exon boundary |
| **111515603** | glutamate receptor ionotropic, kainate 5 | XM_023171815.1 | ATCGGAGTCCAAGACCCTCA | AGGCAGCAGAATAACGCAGT | Within-exon |
| **111515719** | multidrug resistance-associated protein 4-like | XM_023171924.1 | ATTGGGTGTCGTTCTGGACC | TGTGTTCTCTGTAGTTGGGTCG | Spans exon-exon boundary |
| **111515799** | glutamate receptor ionotropic, kainate 1-like | XM_023172001.1 | CGTTGCTACCACACACTGTT | CAAATTGAACTTGATGACACACTTT | Spans exon-exon boundary |
| **111515852** | multidrug resistance protein homolog 49-like | XM_023172055.1 | CTCTCGTGAAGGGCTGGAAA | AGAACTTTGTTGAAACCGTTGT | Spans exon-exon boundary |
| **111515875** | multidrug resistance-associated protein 4-like | XM_023172077.1 | GGCCTATGGTTGGACATTGTG | CAGGTTTCCCATAGTTTTCCATGT | Spans exon-exon boundary |
| **111516142** | venom carboxylesterase-6-like | XM_023172322.1 | AGAGCGGTTCGCCTTTGTTA | TGGATCCAGAGCCTTTCCCA | Within-exon |
| **111516189** | glutamate receptor ionotropic, kainate 2-like | XM_023172364.1 | AGGTAACGCATTCTGGTTTGC | TCCGAGTTGAAGTAGCCTTCG | Spans exon-exon boundary |
| **111516221** | ionotropic receptor 40a | XM_023172399.1 | TCCTCCAGAAAGGACCCAGG | AAAGATCCGCTTCCCTCCTC | Spans exon-exon boundary |
| **111516444** | probable multidrug resistance-associated protein lethal(2)03659 | XM_023172607.1 | TGCCAATGTTGATCCTCAAACT | CCACCGTTAGTACTGTGCAA | Spans exon-exon boundary |
| **111517051** | glutamate receptor ionotropic, kainate 1-like | XM_023173106.1 | AAGCACTGCCACTAAGCTGT | TGCCTTGTGTAGATTTCGATTTCA | Spans exon-exon boundary |
| **111517220** | leucine-rich repeat-containing G-protein coupled receptor 4-like | XM_023173303.1 | CCAGACTCCGCAGTTGGTAA | TCTCCAGATAGTTCATTCCGCT | Within-exon |
| **111517228** | sodium channel protein Nach-like | XM_023173307.1 | TGGTTCGTCTTGGTAGCAGC | GGGGTTTTCCTGATATCTGGTCC | Spans exon-exon boundary |
| **111517372** | major facilitator superfamily domain-containing protein 12-like | XM_023173510.1 | CGGACTTGTTGCATCAATTTGGA | CGAGGATCCACTGATGCCTAA | Spans exon-exon boundary |
| **111517473** | multidrug resistance-associated protein 4-like | XM_023173645.1 | TCGGAAACCATTCGTCACAA | GCACTTTTTAGATGTACCTGATCCA | Spans exon-exon boundary |
| **111517576** | major facilitator superfamily domain-containing protein 6 | XM_023173790.1 | CCGATGTTAATGGTTCAGAATGGT | CAGGAAGGCTGATCTGCTCG | Within-exon |
| **111517753** | probable cytochrome P450 6a23 | XM_023174014.1 | TGTTGTATTCGTATGAGAACCTTTC | ACAGCAGATAACGCTACCAGT | Spans exon-exon boundary |
| **111517755** | probable cytochrome P450 6a23 | XM_023174019.1 | GCCACCCCACTCAAGGGATA | CCAATGTATTTCCAACCTTTTGCC | Spans exon-exon boundary |
| **111517821** | pickpocket protein 28-like | XM_023174098.1 | TGCGTCCAGAATGACCTACG | GGTGGTACTGAATCTCGCTTCT | Spans exon-exon boundary |
| **111518140** | probable cytochrome P450 49a1 | XM_023174495.1 | GGGAGTCATAGGAGTGCATGG | CGGTTGAAGGTGGCAACAAA | Spans exon-exon boundary |
| **111518243** | protein Red | XM_023174636.1 | GCTGAGTGTTATCCAGGTTTGG | ACCAAGATCCATTTTGGAGTAGTCT | Spans exon-exon boundary |
| **111518250** | neuroligin-3-like | XM_023174646.1 | AGGAGACTTCAATCGCGTCC | ATTGTTCAGGAGCACCCCTT | Spans exon-exon boundary |
| **111518298** | cytochrome P450 9e2-like | XM_023174711.1 | AGCGTCAGGTTTCAGACAGT | ACAGCAGCATCGTTTTTATCTTCC | Spans exon-exon boundary |
| **111518309** | acylcarnitine hydrolase-like | XM_023174727.1 | ACTTTAGTTGGACACAGTTCAGGA | CCGCAAATGCGTACTTTGGA | Spans exon-exon boundary |
| **111506516** | obscurin | XM_023161610.1 | ACTACAAGGTCACGGCGAAG | GTGGGTTCCGCTTCCTTGAT | Spans exon-exon boundary |
| **111512141** | A disintegrin and metalloproteinase with thrombospondin motifs 16 | XM_023168241.1 | GACATCGCAGTTGCCATAACA | CCAACGTAGGCAAATCCTGC | Spans exon-exon boundary |
| **111508900** | glutamate-gated chloride channel | XM_023164538.1 | TCTGTCATGTTGCAGCGACT | TTGTTCTGCGGTCTCCAAGG | Spans exon-exon boundary |
| **111515661** | neurexin-1 | XM_023171873.1 | CGGGCAGGATCGACTACAAA | CCAAGGTGGCAGGATCAAGT | Spans exon-exon boundary |
| **111509204** | serine protease gd-like | XM_023164900.1 | GACGTTCTGTGCAGGAGGAA | ACACCATCTATGGGGAGGACT | Spans exon-exon boundary |
| **111502219** | alpha-actinin, sarcomeric (LOC111502219) | XM_023156233.1 | AATGCCGGACGAAAGAGCAG | TGCAGTTTCGGCCTTCTGG | Spans exon-exon boundary |
| **111507845** | coatomer subunit gamma | XM_023163216.1 | AAGACCAGTTCCAGTCTCGC | AGGGGCCCAATTGCTGTATT | Spans exon-exon boundary |
| **111512102** | serine protease nudel | XM_023168198.1 | CGCCGGAGTGGACAATGTAT | ATCGGTAAGGGCAGCACAAT | Spans exon-exon boundary |
| **111510693** | 26S proteasome non-ATPase regulatory subunit 1-like | XM_023166627.1 | TTACATCTGCAGCGGGGATT | TGTCGAGCTTTTTCAAAGCGA | Spans exon-exon boundary |
| **111504672** | titin | XM_023159342.1 | AGGAGGGGTAGAGAACTGCC | TTGGTCCATAACAATCTTTGCG | Spans exon-exon boundary |
| **111518045** | cadherin-related tumor suppressor-like | XM_023174372.1 | AGTCACCATTGAGGTCACCG | TCTCCGGGATGTCGAAGGAA | Spans exon-exon boundary |
| **111517239** | synapsin | XM_023173317.1 | CCAAGTACGACATTCACGTCC | TCGTCTTCCAACAACCTGAGA | Spans exon-exon boundary |
| **111514365** | protein toll-like | XM_023170603.1 | AGCGGCTGAGTGTAGATAACG | GAATCTCATCCTTCCTGCTTCG | Spans exon-exon boundary |
| **111503077** | trissin receptor-like | XM_023157294.1 | GCTTGTGCTTTTTCGGAAACC | GCTTGTGCTTTTTCGGAAACC | Spans exon-exon boundary |
| **111508298** | potassium voltage-gated channel protein Shaw-like | XM_023163746.1 | GCAGATCCTCAATTATTACAGAACA | TCCAATCCCCAGAACTCCAA | Spans exon-exon boundary |
| **111517289** | MOXD1 homolog 2 | XM_023173385.1 | TCAAAGGACGGTAGGATGGC | TGACATGGCGGTCCTGAAAA | Spans exon-exon boundary |
| **111502881** | peptidyl-prolyl cis-trans isomerase | XM_023157046.1 | CGCCAAAATGGTTTTGCCAAG | ACTACATCGTTCCGGAGTTCT | Spans exon-exon boundary |
| **111503672** | neurogenic locus Notch protein | XM_023158033.1 | TGGATGGCATTAACAGTGGTT | TGTCATTCTCACAATGCCTTCC | Spans exon-exon boundary |
| **111512120** | sushi, von Willebrand factor type A, EGF and pentraxin domain-containing protein 1 | XM_023168219.1 | GCAAACGAGACAAGTGAGCA | CCTTGTCGGGAATTTTTGCCA | Spans exon-exon boundary |
| **111514804** | DNA repair protein complementing XP-C cells homolog | XM_023171044.1 | TGGGTGGTAAGTGAAAATGCT | CAGAGGAACTTCATCCATATCCTT | Spans exon-exon boundary |
| **111506459** | rap guanine nucleotide exchange factor 4 | XM_023161526.1 | TGTCCAATTCCGTCAAGCGA | CGCCCTGGTGAAAAAGAACAG | Spans exon-exon boundary |
| **111511138** | probable G-protein coupled receptor Mth-like 1 | XM_023167153.1 | GCTTGCATCCAGACATGGGA | GCGGCCTCAAATCTCTGTCA | Spans exon-exon boundary |
| **111507144** | NFX1-type zinc finger-containing protein 1-like | XM_023162405.1 | TCAAGTTGAATGATACGAGAATGGA | ACTGATCCAAGTGTTCCTTCACA | Spans exon-exon boundary |
| **111518177** | cathepsin L-like | XM_023174539.1 | TGGAGAGAAGTTGGAGCAGT | GCAATCAGGATAACATACGCTGC | Spans exon-exon boundary |
| **111512707** | otopetrin-2-like | XM_023168855.1 | GAAAGCTTACGACTCGCCTG | GATGGGGGTGGACTAGCTTC | Spans exon-exon boundary |
| **111506113** | Down syndrome cell adhesion molecule-like protein Dscam2 | XM_023161064.1 | TGGCTAAAAACACAGAGGGGT | CGATGACACGGCTCTGGTAG | Within-exon |
| **111510619** | ATP-binding cassette sub-family A member 3-like | XM_023166544.1 | CGGAGCGGGTAAAACTACCA | CTTACCGTTAACCCAGCCGT | Spans exon-exon boundary |
| **111501885** | protein gone early\|Leptinotarsa decemlineata protein gone early (LOC111501885)\|zinc metalloprotease family m13 neprilysin-related | XM_023155803.1 | CTGCGCTTTACGGTATGCAA | CTCGAAGTAGTTCACCGCGA | Spans exon-exon boundary |
| **111502009** | vitellogenin receptor-like\|Leptinotarsa decemlineata vitellogenin receptor-like (LOC111502009) | XM_023155954.1 | AACACGGACACCAACTCGAA | ATCCTGCCACTTGTCTGTGC | Spans exon-exon boundary |
| **111504641** | intersectin-1 | XM_023159300.1 | CCAAAACAAGAGGAAAACACTTCC | ATTCCGGCGATTGCAAACTT | Spans exon-exon boundary |
| **111512869** | liprin-beta-1 | XM_023169033.1 | TCTTATCACGCCGTTCCACC | ATCTTGTTCTCCGGGCTTGG | Spans exon-exon boundary |
| **111503227** | cadherin-related tumor suppressor-like | XM_023157478.1 | TGCAATGGACACTGGCTCAC | GGCTTGTCGCTTGAATTCCG | Spans exon-exon boundary |
| **111507712** | low-density lipoprotein receptor-related protein 1B-like | XM_023163059.1 | CGGTAGCATTTTGGACGGAC | CCACATCTGCGTCCCTGTTA | Spans exon-exon boundary |
| **111506705** | head-specific guanylate cyclase | XM_023161878.1 | TGTTTCCTGCTGAGGGTCTG | CCGTCAATGAAAGGCGAACC | Spans exon-exon boundary |
| **111506943** | protein jagged-1b | XM_023162165.1 | ATTGCCATTGTCCGGAAGGT | GCAGCTATCTACACCGTCGT | Spans exon-exon boundary |
| **111505876** | troponin C-like | XM_023160768.1 | CTGGAACTGTTGATTTTGATGAGTT | GAGCTTTCTTGGAGGCGGTA | Spans exon-exon boundary |
| **111518200** | ecdysone-induced protein 78C | XM_023174570.1 | GATTCGACATTCACCAGCGTG | GTGATGGAACCAGGGCTTGA | Spans exon-exon boundary |
| **111517393** | neurogenic protein big brain | XM_023173543.1 | TGTACATTTGCTTCCATGCCG | TCCGAGCCAGTATACCCAGT | Spans exon-exon boundary |
| **111503270** | phosphatidylinositol 3-kinase catalytic subunit type 3 | XM_023157538.1 | GGGACATTTAACTGAATTAAGCGAT | TTCCCTTCCAGTGTTCCTATTTT | Spans exon-exon boundary |
| **111506419** | gastrin/cholecystokinin type B receptor-like | XM_023161484.1 | CGCCCTCTCCAGGAAATTCA | GCTGCTGTCAGCCCACTTA | Spans exon-exon boundary |
| **111507772** | G-protein coupled receptor Mth2-like | XM_023163119.1 | GTGGTTGGCTTTCAGGGGTA | CCCAAGCGTAGCAGCAGTAA | Spans exon-exon boundary |
| **111512535** | actin-3, muscle-specific (B actin) | XM_023168674.1 | CGGTGGATCCATCTTGGCTT | CACTTGCGGTGAACGATTCC | Within-exon |
| **111516602** | actin | XM_023172750.1 | TCCGGACATGGTGTTTCTCG | TTGGTCAGATCTTCACCCGC | Within-exon |
| **111513661** | ADP-ribosylation factor-like protein 1 | XM_023169879.1 | GGTTCCCATGCTAAGGGAAGAA | TGCACCCTGGTATGTCTTGT | Spans exon-exon boundary |
| **111512786** | ADP-ribosylation factor-like protein 4C | XM_023168938.1 | TGTGTGTCACGGATTTTAAGGTG | TCCATTGACTGCTGGTGTGG | Spans exon-exon boundary |
| **111518057** | TATA-box-binding protein-like | XM_023174384.1 | TGTATCAGCTGGTAATTAGGGACT | TTTTCGTCTTCCTGGATTTTGTT | Spans exon-exon boundary |
| **111509024** | TATA-box-binding protein | XM_023164693.1 | TGAGGATACGAGAACCCCGT | GCCAACCTGGAGTCTTCCTC | Within-exon |
| **111505945** | elongation factor 1-alpha | XM_023160856.1 | CCGTCTTCCCCTTCAGGATG | GACAACAACCATACCGGGTT | Spans exon-exon boundary |
| **111511187** | elongation factor 1-beta | XM_023167199.1 | ACAGATGGTGTTTGGATTACCAGA | GGGGGTTAGAGCGGCTTTT | Spans exon-exon boundary |
| **111504191** | elongation factor 2 | XM_023158693.1 | CGAAGCTGCCATTGGAATCA | GCGTCCCTTATCTGAGGTCG | Spans exon-exon boundary |
|  |  |  |  |  |  |
|  |  |  |  |  |  |

**S. Table 4.** Housekeeping Genes

| **GeneID** | **Description** |
| --- | --- |
| 111512535 | actin-3, muscle-specific |
| 111516602 | actin |
| 111513661 | ADP-ribosylation factor-like protein 1 |
| 111512786 | ADP-ribosylation factor-like protein 4C |
| 111518057 | TATA-box-binding protein-like |
| 111509024 | TATA-box-binding protein |
| 111505945 | elongation factor 1-alpha |
| 111511187 | elongation factor 1-beta |
| 111504191 | elongation factor 2 |
| 111510004 | 60S ribosomal protein L4 |
| 111516834 | 60S ribosomal protein L18 |

**S. Figure 1.** *L. decemlineata* Dose Response Probit Regression Analysis to Imidacloprid and Isocycloseram

**S. Figure 1.** Individual probit curves showing percent mortality (proportion harmed/incapacitated) plotted against log10-transformed concentrations (ppm) for all tested populations at 48 and 96 hours post-exposure. Orange curves represent imidacloprid responses and blue curves represent isocycloseram responses. Observed data points are overlaid on fitted probit models. Vertical dashed lines indicate LC20, LC50, and LC90 estimates for each population-insecticide combination. Population codes: AARS (WI Arlington, 1^st^ and 2^nd^ generation), HARS (WI Hancock, 1^st^ and 2^nd^ generation), MD (Maryland), ME (Maine), NC (North Carolina), ND (North Dakota), NY (New York), OR (Oregon), VA (Virginia).

**S. Table 5.** Differentially Expressed Genes by Treatment

| **Gene_ID** | **baseMean** | **log2FoldChange** | **lfcSE** | **stat** | **pvalue** | **padj** | **Sequence** | **Description** | **treatment** |
| --- | --- | --- | --- | --- | --- | --- | --- | --- | --- |
| 111501956 | 1261.71778562457 | 0.0103383046125322 | 0.0026840144916288 | 3.85180655498558 | 0.0001172496312056 | 0.0020181938913528 | XM_023155891.1 | Leptinotarsa decemlineata disintegrin and metalloproteinase domain-containing protein 11 (LOC111501956) | Control |
| 111503224 | 5186.61776300008 | -0.0053218262728215 | 0.0017479878129411 | -3.04454426593926 | 0.0023303313402923 | 0.0167783856501048 | XM_023157473.1 | major facilitator superfamily domain-containing protein 6-B | Control |
| 111503227 | 1890.60218509347 | 0.0105121062656719 | 0.0030048124912967 | 3.49842337787116 | 0.0004680175450177 | 0.0048138947487543 | XM_023157478.1 | cadherin-related tumor suppressor-like | Control |
| 111504404 | 1645.0560568941 | 0.0056818115405901 | 0.0019690551105797 | 2.8855523190091 | 0.0039072751237939 | 0.0222752815558163 | XM_023158946.1 | transient receptor potential cation channel protein painless-like | Control |
| 111506113 | 137.551518456651 | 0.0103141860547722 | 0.0032826013433337 | 3.1420769615287 | 0.001677539349055 | 0.015097854141495 | XM_023161064.1 | Down syndrome cell adhesion molecule-like protein Dscam2 | Control |
| 111508973 | 3856.62795452805 | 0.0104035220178279 | 0.0037894228310352 | 2.74541070809609 | 0.0060435211162861 | 0.0290089013581735 | XM_023164631.1 | voltage-dependent T-type calcium channel subunit alpha-1G-like | Control |
| 111509024 | 2284.35103973987 | -0.0104338142842936 | 0.0016491006201699 | -6.32697250651576 | 2.50017880203677E-10 | 1.80012873746648E-08 | XM_023164693.1 | TATA-box-binding protein | Control |
| 111509258 | 6734.25422186158 | -0.0059635157921103 | 0.0019411740886353 | -3.07211796562911 | 0.0021254570987665 | 0.0167783856501048 | XM_023164970.1 | glutamate receptor ionotropic, kainate 2-like | Control |
| 111511127 | 4354.14716715127 | 0.0052198937209551 | 0.0018296365708261 | 2.85296752600335 | 0.0043313047469642 | 0.0222752815558163 | XM_023167144.1 | voltage-dependent calcium channel type A subunit alpha-1 | Control |
| 111511138 | 1396.00924545025 | 0.012607950573935 | 0.0030837979559407 | 4.08844896911831 | 4.34267050978936E-05 | 0.0010422409223494 | XM_023167153.1 | probable G-protein coupled receptor Mth-like 1 | Control |
| 111511268 | 182.888054420724 | -0.0090223611304225 | 0.0023693802174457 | -3.80789924048107 | 0.0001401523535661 | 0.0020181938913528 | XM_023167286.1 | cytochrome P450 307a1-like | Control |
| 111513883 | 1137.31749055218 | -0.008949362383016 | 0.0035034396711291 | -2.55445026120052 | 0.0106355623825018 | 0.0450447347964782 | XM_023170104.1 | venom carboxylesterase-6-like | Control |
| 111514255 | 2461.18354000615 | 0.0053585436280834 | 0.001865716420921 | 2.87211044936734 | 0.0040774043252175 | 0.0222752815558163 | XM_023170493.1 | voltage-dependent calcium channel type A subunit alpha-1-like | Control |
| 111514427 | 111.998027619416 | -0.0101855082103445 | 0.003902162096326 | -2.61022170758473 | 0.0090483559392955 | 0.0407176017268298 | XM_023170669.1 | transient receptor potential cation channel subfamily V member 5 | Control |
| 111515214 | 8049.35125104688 | -0.0101140429321754 | 0.0018049152365424 | -5.60361103247714 | 2.09931529687741E-08 | 7.55753506875869E-07 | XM_023171444.1 | glutathione S-transferase 1-like | Control |
| 111515375 | 11943.0538085728 | -0.0076318354324506 | 0.0025328515920398 | -3.01313959982322 | 0.0025855994848284 | 0.0169239239006955 | XM_023171602.1 | glutathione S-transferase 1-like | Control |
| 111518045 | 1163.39931678103 | 0.0105552411730129 | 0.0029057775542247 | 3.6325014479056 | 0.0002806870005266 | 0.0033682440063194 | XM_023174372.1 | cadherin-related tumor suppressor-like | Control |
| 111502194 | 1395.909738016 | 0.0082643766655109 | 0.0022276425173952 | 3.7099205105738 | 0.0002073243317807 | 0.0024101453569508 | XM_023156204.1 | multidrug resistance-associated protein 4-like (LOC111502194) | Imidacloprid |
| 111503276 | 1563.16592872081 | 0.0094252118412005 | 0.0027781551042625 | 3.39261541831821 | 0.0006922875976026 | 0.0058529769615498 | XM_023162359.1 | voltage-dependent L-type calcium channel subunit beta-2 | Imidacloprid |
| 111503672 | 272.578684123339 | 0.0082915898476624 | 0.0024363374872626 | 3.40330101679727 | 0.000665768875611 | 0.0058529769615498 | XM_023158033.1 | neurogenic locus Notch protein | Imidacloprid |
| 111503873 | 1926.00641904007 | 0.0077974135762092 | 0.0029819730222632 | 2.61485047584074 | 0.0089266524697227 | 0.0377374651655535 | XM_023158303.1 | sodium channel protein 60E-like | Imidacloprid |
| 111505188 | 2755.47341915313 | 0.0119709328182733 | 0.003668148815141 | 3.26348068782294 | 0.0011005268443538 | 0.0066885172106721 | XM_023159960.1 | voltage-dependent T-type calcium channel subunit alpha-1G-like | Imidacloprid |
| 111506324 | 1785.26972444558 | 0.0075030993536727 | 0.002406006933904 | 3.11848617223143 | 0.0018178267171587 | 0.0099445814526921 | XM_023161359.1 | muscle calcium channel subunit alpha-1-like | Imidacloprid |
| 111506459 | 2909.24307490368 | 0.0103198091116021 | 0.0024216809318294 | 4.26142394564184 | 2.03128482758322E-05 | 0.000472273722413 | XM_023161526.1 | rap guanine nucleotide exchange factor 4 | Imidacloprid |
| 111506705 | 44.4738449961985 | 0.0133127570767265 | 0.0034424563821466 | 3.86722607315218 | 0.000110080369965 | 0.0015010964080941 | XM_023161878.1 | head-specific guanylate cyclase | Imidacloprid |
| 111506943 | 1469.3427471043 | 0.0132897890478481 | 0.0026264903270023 | 5.05990405188961 | 4.19467501534458E-07 | 3.90104776427046E-05 | XM_023162165.1 | protein jagged-1b | Imidacloprid |
| 111506979 | 904.815357847644 | 0.0114051254003071 | 0.0026753384692252 | 4.26305887329832 | 2.01647362738784E-05 | 0.000472273722413 | XM_023162201.1 | glutamate [NMDA] receptor subunit 1-like | Imidacloprid |
| 111507099 | 5570.77923072626 | -0.0066778189376315 | 0.001998531486262 | -3.34136288746762 | 0.0008336818193793 | 0.0059640314770987 | XM_023162347.1 | cytochrome P450 9e2-like | Imidacloprid |
| 111508023 | 382.599401746812 | -0.0063901710726575 | 0.002443817317514 | -2.61483173347754 | 0.0089271422972277 | 0.0377374651655535 | XM_023163440.1 | soluble guanylate cyclase 89Db-like | Imidacloprid |
| 111509532 | 5003.31458715335 | -0.0051558272190736 | 0.0019257051642107 | -2.67737103004899 | 0.0074202413563007 | 0.0359140443670092 | XM_023165296.1 | glutathione S-transferase 1-like | Imidacloprid |
| 111509814 | 292.070385346978 | -0.0123219696529902 | 0.0037400891791046 | -3.29456573437637 | 0.0009857388633869 | 0.0065481224496419 | XM_023165644.1 | glutathione S-transferase D2-like | Imidacloprid |
| 111510198 | 4244.67217299868 | -0.004378207517662 | 0.0017272864699937 | -2.53473155363618 | 0.0112533526258885 | 0.0455026867046797 | XM_023166095.1 | multidrug resistance protein homolog 49-like | Imidacloprid |
| 111510322 | 2371.18658190363 | 0.0076630014949351 | 0.0016749090679064 | 4.57517464187694 | 4.75822897893505E-06 | 0.0002212576475204 | XM_023166225.1 | major facilitator superfamily domain-containing protein 12-like | Imidacloprid |
| 111511764 | 1799.23455458485 | 0.0065075516527442 | 0.0024428423145586 | 2.66392620348887 | 0.0077234504015073 | 0.0359140443670092 | XM_023167793.1 | voltage-dependent calcium channel subunit alpha-2/delta-3 | Imidacloprid |
| 111511812 | 876.886943048746 | 0.0090296590907272 | 0.0025403662053534 | 3.55447142687489 | 0.0003787396625433 | 0.0039136431796143 | XM_023167829.1 | ATP-binding cassette sub-family A member 3-like | Imidacloprid |
| 111512003 | 8625.66925742909 | -0.0107533090820474 | 0.0025776362549524 | -4.17177135113104 | 3.02240845812526E-05 | 0.0005621679732112 | XM_023168093.1 | multidrug resistance-associated protein 4-like | Imidacloprid |
| 111515661 | 1059.58036060598 | 0.0138763836392108 | 0.0035941114786802 | 3.86086623120169 | 0.0001129857511468 | 0.0015010964080941 | XM_023171873.1 | neurexin-1 | Imidacloprid |
| 111515719 | 4044.5096730726 | 0.0061741641931433 | 0.0022424373875834 | 2.75332735144812 | 0.0058992876442467 | 0.0304796528286082 | XM_023171924.1 | multidrug resistance-associated protein 4-like | Imidacloprid |
| 111518200 | 1116.64852477883 | 0.0094762565147953 | 0.0029150325948111 | 3.25082351794735 | 0.0011507126383952 | 0.0066885172106721 | XM_023174570.1 | ecdysone-induced protein 78C | Imidacloprid |
| 111518243 | 8199.82844437072 | -0.0064401259310892 | 0.001926853633978 | -3.34230157263868 | 0.0008308673666352 | 0.0059640314770987 | XM_023174636.1 | protein Red | Imidacloprid |
| 111502371 | 152.215125412462 | 0.0101000061621525 | 0.0022830117500697 | 4.42398343409493 | 9.68974535921676E-06 | 0.0001792602891455 | XM_023156445.1 | glutamate receptor 1 | Isocycloseram |
| 111502767 | 44.5211799859151 | 0.017124485204279 | 0.0056554304684599 | 3.02797201729939 | 0.002462008840201 | 0.017080186328895 | XM_023156918.1 | multidrug resistance-associated protein 4-like | Isocycloseram |
| 111504198 | 3295.05234143494 | 0.0097428057531821 | 0.0025326011686202 | 3.84695619424752 | 0.000119594342007 | 0.0014749968847531 | XM_023158701.1 | protein tramtrack, beta isoform | Isocycloseram |
| 111504218 | 941.165597867346 | -0.0133563547663571 | 0.0033003608876952 | -4.04693765950077 | 5.18920541057981E-05 | 0.0007200022507179 | XM_023158724.1 | cytochrome P450 4c3-like | Isocycloseram |
| 111505753 | 1791.11676736934 | 0.0062628807708571 | 0.0023945418551101 | 2.61548185407225 | 0.0089101655584788 | 0.0458752430069068 | XM_023160617.1 | venom carboxylesterase-6-like | Isocycloseram |
| 111505876 | 5746.57265800658 | -0.0110806686198323 | 0.0025705084457385 | -4.31069138800222 | 1.62744903023447E-05 | 0.0002580669176514 | XM_023160768.1 | troponin C-like | Isocycloseram |
| 111505945 | 9525.97924411543 | -0.0091349852659927 | 0.0035019256173548 | -2.60856062182517 | 0.0090923905058734 | 0.0458752430069068 | XM_023160856.1 | elongation factor 1-alpha | Isocycloseram |
| 111507261 | 236.640595940444 | -0.0150172004937261 | 0.0040878305324192 | -3.67363577688201 | 0.0002391235197478 | 0.0023991489538969 | XM_023162551.1 | cholinesterase-like | Isocycloseram |
| 111508711 | 260.54055505079 | 0.0071351117757252 | 0.0025267099563124 | 2.82387448464341 | 0.0047446961632956 | 0.0306552269124432 | XM_023164304.1 | cytochrome P450 315a1, mitochondrial | Isocycloseram |
| 111508880 | 2725.69143762073 | -0.0184185051315965 | 0.0023375472050179 | -7.87941526573592 | 3.28916657083022E-15 | 3.65097489362154E-13 | XM_023164506.1 | cytochrome P450 6a2-like | Isocycloseram |
| 111508881 | 5010.92113184662 | -0.0080796828731546 | 0.0029449160007282 | -2.74360384851612 | 0.0060768823652001 | 0.0337266971268607 | XM_023164508.1 | cytochrome P450 6a2-like | Isocycloseram |
| 111508891 | 5596.99944679278 | -0.010491189934364 | 0.0028282172335168 | -3.70947104417379 | 0.000207692707633 | 0.0023053890547269 | XM_023164524.1 | esterase E4-like | Isocycloseram |
| 111512274 | 751.032702829942 | -0.0118976432316118 | 0.0035068154784092 | -3.3927200632207 | 0.0006920232038527 | 0.0059088135098194 | XM_023168378.1 | liver carboxylesterase 1-like | Isocycloseram |
| 111512344 | 206.792986550406 | -0.0103867192910887 | 0.0028434730112536 | -3.6528285128718 | 0.0002593674544753 | 0.0023991489538969 | XM_023168452.1 | sodium channel protein Nach-like | Isocycloseram |
| 111513747 | 5505.13362934175 | -0.0107631680913561 | 0.0019614614110196 | -5.48732084704194 | 4.0807553772389E-08 | 1.88739537628546E-06 | XM_023169972.1 | cytochrome P450 6k1-like | Isocycloseram |
| 111513755 | 1883.66648671816 | -0.0114857185555055 | 0.002488734059649 | -4.61508472991494 | 3.92934976580527E-06 | 8.7231564800877E-05 | XM_023169981.1 | acetylcholine receptor subunit beta-like 2 | Isocycloseram |
| 111515003 | 608.585411858853 | -0.0177129750138422 | 0.0034068547650851 | -5.19921635502994 | 2.00130470205164E-07 | 5.5536205481933E-06 | XM_023171236.1 | probable multidrug resistance-associated protein lethal(2)03659 | Isocycloseram |
| 111515122 | 8282.16043156646 | -0.0069495900188607 | 0.0022022372006032 | -3.15569549772254 | 0.0016011592332909 | 0.011848578326353 | XM_023171357.1 | glutathione S-transferase 1 | Isocycloseram |
| 111515157 | 625.981540940605 | -0.0105107289256504 | 0.003741938551145 | -2.80889939318591 | 0.0049711178776934 | 0.0306552269124432 | XM_023171390.1 | sodium channel protein Nach-like | Isocycloseram |
| 111517289 | 4385.51414605581 | 0.0069271255179091 | 0.0025076165615014 | 2.76243410745436 | 0.0057372140932797 | 0.0335174086502133 | XM_023173385.1 | MOXD1 homolog 2 | Isocycloseram |
| 111517372 | 1646.83076048153 | -0.0114505170324783 | 0.0021018796415536 | -5.4477510539159 | 5.10106858455528E-08 | 1.88739537628546E-06 | XM_023173510.1 | major facilitator superfamily domain-containing protein 12-like | Isocycloseram |
| 111517473 | 1.94796118690358 | -0.0434259584925172 | 0.0130746896220441 | -3.32137586037226 | 0.000895748199808 | 0.0071020035841921 | XM_023173645.1 | multidrug resistance-associated protein 4-like | Isocycloseram |

**S. Table 6.** PERMANOVA Data

| **Population** | **Comparison** | **R²** | **F-value** | **p-value** | **Significance** |
| --- | --- | --- | --- | --- | --- |
| All | Treatment × Population | 0.369 | 14.92 | <0.001 | *** |
| AARS | Treatment | 0.098 | 0.97 | 0.407 | ns |
| HARS | Treatment | 0.536 | 13.31 | <0.001 | *** |
| MD | Treatment | 0.564 | 16.81 | <0.001 | *** |
| ME | Treatment | 0.675 | 31.22 | <0.001 | *** |
| ND | Treatment | 0.63 | 20.4 | <0.001 | *** |
| NY | Treatment | 0.366 | 8.94 | <0.001 | *** |
| OR | Treatment | 0.575 | 20.25 | <0.001 | *** |
| VA | Treatment | 0.196 | 3.04 | 0.019 | * |
| NC | – | – | – | skipped | – |
| AARS | ISM Dose | 0.001 | 0.01 | 0.992 | ns |
| HARS | ISM Dose | 0.384 | 9.97 | 0.006 | ** |
| MD | ISM Dose | 0.041 | 0.64 | 0.542 | ns |
| ME | ISM Dose | 0.064 | 1.1 | 0.336 | ns |
| ND | ISM Dose | 0.085 | 1.49 | 0.238 | ns |
| NY | ISM Dose | 0.062 | 1.06 | 0.323 | ns |
| OR | ISM Dose | 0.039 | 0.76 | 0.517 | ns |
| VA | ISM Dose | 0.062 | 1.05 | 0.33 | ns |
| AARS | Imidacloprid Dose | 0.132 | 1.07 | 0.271 | ns |
| HARS | Imidacloprid Dose | 0.182 | 2.68 | 0.058 | • |
| MD | Imidacloprid Dose | 0.127 | 2.32 | 0.131 | ns |
| ME | Imidacloprid Dose | 0.297 | 8.01 | 0.008 | ** |
| ND | Imidacloprid Dose | 0.229 | 3.85 | 0.061 | • |
| NY | Imidacloprid Dose | 0.091 | 1.99 | 0.149 | ns |
| OR | Imidacloprid Dose | 0.129 | 2.81 | 0.052 | • |
| VA | Imidacloprid Dose | 0.153 | 2.53 | 0.068 | • |

**S. Table 7.** Probit regression estimates of lethal concentrations (LC_x_) for L. decemlineata populations exposed to Imidacloprid and Isocycloseram

|  |  | **LC_x_ (ppm) ± (95% CI) for Imidacloprid** | | **LC_x_ (ppm) ± (95% CI) for Isocycloseram** | |
| --- | --- | --- | --- | --- | --- |
| **Population** | **LC_x_** | **48h** | **96h** | **48h** | **96h** |
| **AARS** | **20**  **50**  **90**  **RR_50_**  ***X^2^***  ***Slope*** | 0.0949 (0.012-0.778)  2.83 (0.914-8.756)  497 (44.421-5558.323)  10.066  *32.0578*  *0.5709* | 0.0646 (0.007-0.577)  2.24 (0.683-7.376)  498 (36.014-6874.829)  -  *25.5479*  *0.5463* | 0.362 (0.208-0.631)  1.53 (0.917-2.55)  13.7 (6.9-28.8)  25.929  *37.1846*  *1.3464* | *0.0817 (0.037-0.182)  *0.933 (0.416-2.09)  *38 (6.55-220)  -  *66.0098*  *0.7959* |
| **AARS (2nd gen)** | **20**  **50**  **90**  **RR_50_**  ***X^2^***  ***Slope*** | 0.607 (0.172-2.139)  6.31 (3.297-12.057)  222 (77.639-637.389)  11.237  *27.4232*  *0.88431* | 0.364 (0.126-1.051)  3.26 (1.778-5.977)  91.7 (34.651-242.856)  -  *28.4210*  *0.8843* | *0.0122 (0.005-0.029)  *0.223 (0.086-0.58)  *18.7 (1.52-232)  31.002  *67.4515*  *0.6660* | 0.0514 (0.035-0.076)  0.267 (0.164-0.435)  3.28 (1.12-9.63)  -  *54.9876*  *1.1761* |
| **HARS** | **20**  **50**  **90**  **RR_50_**  ***X^2^***  ***Slope*** | 0.103 (0.003-3.072)  15.7 (2.995-82.160)  33000 (678.466-1608134)  26.726  *51.6739*  *0.3856* | 0.0071 (3.60E-04-0.138)  1.12 (0.225-5.600)  2530 (42.041-152067.348)  -  *47.9075*  *0.3822* | 0.482 (0.222-1.05)  2.82 (1.44-5.51)  41.4 (15.3-112)  47.809  *45.5662*  *1.0980* | 0.192 (0.089-0.414)  1.61 (0.769-3.36)  40.9 (10.3-163)  -  *55.2763*  *0.9116* |
| **HARS (2nd gen)** | **20**  **50**  **90**  **RR_50_**  ***X^2^***  ***Slope*** | 0.00847 (0.001-0.069)  0.582 (0.173-1.960)  365 (16.774-7932.245)  1.092  *46.5776*  *0.4581* | 0.0118 (0.002-0.070)  0.549 (0.191-1.579)  189 (13.482-2647.900)  -  *49.3693*  *0.5051* | *0.0367 (8.53E-04-1.58)  *13.9 (0.381-503)  *116000 (9.96-1360000)  25.998  *104.1965*  *0.3266* | *4.12E-05 (4.36E-09-0.39)  *0.817 (1.17E-04-5730)  *2850000 (3.23E-05-2.52E+17)  -  *68.9091*  *0.1958* |
| **MD** | **20**  **50**  **90**  **RR_50_**  ***X^2^***  ***Slope*** | 0.204 (0.051-0.814)  3.46 (1.633-7.311)  258 (60.539-1097.514)  12.004  *53.4241*  *0.6843* | 0.268 (0.042-1.698)  7.49 (2.987-18.772)  *1190 (184.352-7728.024)  -  *57.9992*  *0.5818* | 0.0335 (0.025-0.046)  0.154 (0.099-0.239)  1.56 (0.545-4.49)  2.631  *39.2608*  *1.271* | 0.0204 (0.016-0.026)  0.0783 (0.053-0.116)  0.607 (0.219-1.68)  -  *31.2754*  *1.4409* |
| **ME** | **20**  **50**  **90**  **RR_50_**  ***X^2^***  ***Slope*** | 9.83 (0.259-372.364)  428 (99.572-1838.459)  134000 (7401.971-2423388)  58.575  *35.6589*  *0.5135* | 2.65 (0.027-260.953)  436 (59.079-3223.461)  *1030000 (12191.504-81072577)  -  *64.3037*  *0.3797* | 0.165 (0.077-0.354)  1.44 (0.686-3.03)  39.2 (9.38-164)  24.483  *60.3178*  *0.8932* | 0.0411 (0.008-0.188)  1.62 (0.365-7.21)  *438 (11.1-17200)  -  *88.3829*  *0.5270* |
| **NC** | **20**  **50**  **90**  **RR_50_**  ***X^2^***  ***Slope*** | 1.22 (0.296-5.003)  10.9 (5.064-23.408)  306 (112.043-837.555)  23.163  *40.0426*  *0.8842* | 1.98 (0.299-13.053)  25.2 (9.438-67.366)  *1220 (317.090-4669.209)  -  *53.7787*  *0.7612* | 0.0092 (0.003-0.031)  0.282 (0.082-0.97)  *51 (1.89-1370)  2.631  *118.6897*  *0.5676* | 0.0298 (0.015-0.059)  0.33 (0.157-0.694)  12.9 (2.13-78)  -  *84.9647*  *0.8053* |
| **ND** | **20**  **50**  **90**  **RR_50_**  ***X^2^***  ***Slope*** | 0.0202 (0.001-0.296)  2.18 (0.527-9.036)  2720 (83.591-88772.543)  7.550  *35.4186*  *0.4139* | 0.0026 (8.68E-05-0.079)  0.658 (0.103-4.217)  2980 (23.018-385198.068)  -  *36.5387*  *0.3505* | 0.482 (0.2-1.16)  3.41 (1.6-7.31)  67.4 (20.6-221)  57.955  *48.0139*  *0.9893* | 0.0573 (0.019-0.174)  1.2 (0.397-3.61)  *122 (9.21-1620)  -  *66.2668*  *0.6378* |
| **NY** | **20**  **50**  **90**  **RR_50_**  ***X^2^***  ***Slope*** | 25.5 (0.714-908.985)  629 (164.001-2411.519)  83000 (5878.588-1171224)  62.361  *26.4360*  *0.6043* | 7.59 (0.697-82.759)  118 (45.661-302.419)  7610 (1337.881-43316.80)  -  *49.1588*  *0.7074* | 1.97E-04 (8.22E-06-0.005)  0.0653 (0.003-1.54)  449 (0.049-4130000)  145.824  *35.8931*  *0.3339* | 0.0806 (0.013-0.501)  4.13 (0.718-23.7)  *1650 (24.7-111000)  -  *99.7204*  *0.4923* |
| **OR** | **20**  **50**  **90**  **RR_50_**  ***X^2^***  ***Slope*** | 3.97 (0.779-20.184)  36 (18.132-71.382)  1030 (336.029-3178.914)  34.744  *26.6181*  *0.8787* | *0.05 (0.021-0.121)  *0.559 (0.294-1.064)  *22.1 (5.556-87.799)  -  *64.2012*  *0.8026* | 0.119 (0.087-0.163)  0.402 (0.268-0.603)  2.57 (1.22-5.4)  6.610  *45.5973*  *1.5914* | 0.0798 (0.054-0.119)  0.381 (0.236-0.615)  4.12 (1.56-10.9)  -  *55.0427*  *1.2400* |
| **VA** | **20**  **50**  **90**  **RR_50_**  ***X^2^***  ***Slope*** | 0.219 (0.083-0.576)  1.95 (1.070-3.558)  54.6 (19.704-151.509)  6.459  *38.2351*  *0.8855* | 0.196 (0.076-0.502)  1.73 (0.950-3.145)  47.8 (17.064-133.619)  -  *32.8809*  *0.8892* | 0.201 (0.123-0.329)  0.91 (0.55-1.5)  9.06 (3.9-21.1)  15.445  *46.2885*  *1.2837* | 0.0899 (0.046-0.176)  0.783 (0.391-1.57)  21.1 (4.93-90.6)  -  *60.3515*  *0.8952* |

LCₓ values (ppm) with 95% confidence intervals (CI) given in parentheses for imidacloprid and isocycloseram at two exposure times (48 h and 96 h) across multiple *L. decemlineata* populations^[1]^. LC_20_, LC_50_, and LC_90_ values estimate the concentrations of insecticide required to kill 20%, 50%, and 90% of individuals, respectively. Also shown are chi-square goodness-of-fit (X²) statistics and slope values for each probit regression. LC values from models with poor fit (χ² test, p < 0.05) were flagged but retained in the analysis. AARS = Arlington Agricultural Research Station, Wisconsin, HARS = Hancock Agricultural Research Station, Wisconsin, MD = Maryland, ME = Maine, NC = North Carolina, ND = North Dakota, NY = New York, OR = Oregon, VA = Virginia
